# Detection of Quantitative Trait *Loci* Controlling the Content of Phenolic Compounds in an Asian Plum (*Prunus salicina* L.) F1 Population

**DOI:** 10.3389/fpls.2021.679059

**Published:** 2021-07-09

**Authors:** Diego Valderrama-Soto, Juan Salazar, Ailynne Sepúlveda-González, Claudia Silva-Andrade, Claudio Gardana, Héctor Morales, Benjamin Battistoni, Pablo Jiménez-Muñoz, Mauricio González, Álvaro Peña-Neira, Rodrigo Infante, Igor Pacheco

**Affiliations:** ^1^Instituto de Nutrición y Tecnología de Alimentos (INTA), Universidad de Chile, Santiago, Chile; ^2^Department of Plant Breeding, Centro de Edafología y Biología Aplicada del Segura, Consejo Superior de Investigaciones Científicas (CEBAS-CSIC), Murcia, Spain; ^3^Laboratorio de Biología de Redes, Centro de Genómica y Bioinformática, Facultad de Ciencias, Universidad Mayor, Santiago, Chile; ^4^Dipartimento di Scienze per gli Alimenti, la Nutrizione, l'Ambiente, Università degli Studi di Milano, Milan, Italy; ^5^Departamento de Agroindustria y Enología, Universidad de Chile, Santiago, Chile; ^6^Programa de Doctorado en Ciencias Silvoagropecuarias y Veterinarias, Campus Sur Universidad de Chile, Santiago, Chile; ^7^Departamento de Producción Agrícola, Universidad de Chile, Santiago, Chile; ^8^Center for Genome Regulation (CGR), Santiago, Chile

**Keywords:** Quantitative Trait *Loci*, phenolic compounds, *Prunus salicina*, antioxidant activity, flavonoids, procyanidins, anthocyanins, BLUP

## Abstract

Consumption of fresh fruit is known to protect against non-communicable diseases due to the fruit's content in compounds with an antioxidant capacity, among them is polyphenols. Asian plums (*Prunus salicina L*.) accumulate more than 40 phenolic compounds, with a remarkable diversity in their profiles, depending on the variety and environmental conditions. Although candidate genes have been indicated to control this trait, the *loci* controlling its phenotypic variation have not yet been defined in this species. The aim of this work was to identify the quantitative trait *Loci* (QTL) controlling the phenolic compounds content in the Asian plum skin and flesh. Using UHPLC-DAD-Orbitrap-MS, we determined that cyanidin-3-glucoside and cyanidin-3-rutinoside are the main anthocyanins in Asian plums. Other anthocyanins found to a lesser extent were tentatively identified as cyanidin bound to different sugar and procyanidin moieties. Then we phenotyped fruits of 92 and 80 F1 seedlings from the cross < “98.99” × “Angeleno”> (98 Ang) for two harvest seasons. We used HPLC-DAD to quantify single anthocyanin and spectrophotometric techniques to determine the total content of phenols, flavonoids, procyanidins, and antioxidant activity (DPPH and FRAP). To determine the phenotype-genotype association of phenolic compounds content, phenotypic values (adjusted by linear mixed-effects models), genotypic data and linkage maps were analyzed with the multiple QTL model (MQM) approach. We found a total of 21 significant trait-marker associations: 13 QTLs segregating from “98.99” and 8 QTLs from “Angeleno.” From these associations, 8 corresponded to phenolic compound content in the flesh and 13 in the skin. Phenotype variance was explained by the detected *loci*, ranging from 12.4 to 27.1%. The identified *loci* are related to the content of cyanidin-3-glucoside (LG4), cyanidin-3-rutinoside (LG4), total flavonoids and procyanidins (LG5 and LG8), and minor anthocyanin compounds (LG3 and LG4). These results will help improve the efficiency of breeding programs for the generation of Asian plum varieties with high phenolic compound content.

## Introduction

Consumption of fresh fruits and vegetables is essential for healthy nutrition since they constitute notable sources of phytonutrients and bioactive compounds, in addition to the energy and fiber intake (Pereira and Ludwig, 2001; Shulaev et al., 2008; WHO, 2018). Among these components, phenolic compounds are highlighted as one of the most numerous and ubiquitous secondary metabolites in the plant kingdom (Naczk and Shahidi, 2004). Phenolic compounds are produced in different organs and are associated with several processes, such as tissue pigmentation (Howell, 1974; Konczak-Islam et al., 2003), the resistance to biotic and abiotic stress (Howell, 1974; Beckman, 2000; Jeandet et al., 2002), the attraction of pollinators (Jakubska et al., 2005; Zhang et al., 2016), and are essential components of the cell wall (Naczk and Shahidi, 2004; Ogah et al., 2014). These compounds are characterized by having at least one aromatic ring attached to one or more hydroxyl groups, allowing an electron to be delivered to the reactive oxygen species (ROS), thus neutralizing them. This structural conjugation gives these compounds antioxidant properties (Balsano and Alisi, 2009), leading to great interest in these compounds in recent years. The daily consumption of fruit with a high content of phenolic compounds is strongly related to human health benefits since they exert a protective quality against diseases associated with cellular oxidative stress and aging, such as cardiovascular diseases, diabetes, and Parkinson's, among others (Haminiuk et al., 2012; Jaiswal et al., 2013; Verdu et al., 2014).

The Rosaceae family includes around 100 genera and ~3,000 species, with members of commercial importance such as plums, apples, peaches, almonds, pears, and cherries, among others (Shulaev et al., 2008; Ogah et al., 2014). The fruits of these species contain high richness and diversity in phenolic compounds that, in addition to the previously mentioned nutritional attributes, are responsible for organoleptic properties, such as bitterness, astringency, color, and aroma (Shulaev et al., 2008; Verdu et al., 2014). Flavonoids are among the most important polyphenols in fruits and vegetables, including several subgroups such as flavonols, flavan-3-ols, anthocyanins, isoflavones, dihydrochalcones, flavones, flavononols, and flavanones. The first five are present in rosaceous fruits (Ogah et al., 2014). Anthocyanins are one of the most named flavonoid subgroups due to their health benefits (antioxidant, anti-inflammatory, and antidiabetic activity) and relation to fruit color (Garzón, 2008; González et al., 2016a).

The Asian plum (*Prunus salicina* L.) is an economically important fruit tree species worldwide. According to FAOSTAT Database (2019), the category “plums and sloes” reached an estimated harvested area of 2,727,745 ha. Cultivars of Asian plums show significant phenotypic diversity in the content and profile of phenolic compounds, containing more than 40 different molecules (Jaiswal et al., 2013). For this reason, the Asian plum is an excellent target for the study and dissection of the genetic mechanisms controlling this variability, knowing such mechanisms is crucial to generate tools to improve the efficiency of breeding methods associated with human health benefits.

Phenolic compounds are biosynthesized in the phenylpropanoid and flavonoid pathways. Transcription patterns of genes involved in these pathways correlate to the evolution of content of different phenolic compounds throughout the fruit ripening process. Such patterns are highly dependent on the cultivar (González et al., 2016a,b), leading to high diversity in the content of phenolic compounds. MYB transcription factors appear as master regulators of anthocyanin and procyanidin biosynthesis. The anthocyanin content and the expression of several structural genes for their biosynthesis correlate positively with MYB10 and negatively with MYB1 (González et al., 2016b). In turn, Cheng et al. (2016) showed that the expression of PsMYB10 was induced by ethylene and suppressed by 1-MCP. Also, the PsMYB10 expression was correlated with ethylene signaling pathway-associated genes and some anthocyanin biosynthetic genes (Cheng et al., 2016). Furthermore, El-Sharkawy et al. (2009) observed a correlation of ethylene with anthocyanin accumulation, the expression of seven structural genes (PAL, CHS, CHI, F3H, FDR, LDOX/ANS, and UFGT), and the expression of the MYB10 transcription factor, which was involved in the initial stage of the anthocyanin biosynthetic pathway. These data indicate that the master regulators of flavonoid biosynthesis are controlled by ethylene and suggest that the sources of variation in the flavonoid content in plums, depending on the genetic background, can participate in various steps of the ripening process. In peach, MYB10 genes have been reported as significant regulators of skin anthocyanin biosynthesis through fruit ripening, with a codominance effect of the alleles in this locus (Tuan et al., 2015). In the blood-fleshed peach varieties, the expression of the structural genes UDP-glucose flavonoid 3-O-glucosyltransferase (UGFT) and both leucoanthocyanidin reductase (LAR) and anthocyanidin reductase (ANR) were significantly correlated with the accumulation of cyanidin-3-glucoside and (-)-epicatechin, respectively (Yan et al., 2017).

The content of phenolic compounds in the Asian plums (as in other species) is a quantitative trait since it depends on the variety (genetic component) and the environmental conditions (Venter et al., 2013, 2014; González et al., 2016a). Quantitative genetic mapping is a powerful approach to evaluate the participation of a *locus* in the genetic control of a trait in a genomic context and across environments. QTL mapping has given valuable results about genetic markers associated with a phenotype, providing positive implications for marker-assisted selection (MAS) and genomic dissection of several quantitative traits, including traits related to the synthesis of health-promoting compounds (Collard et al., 2005). Shen et al. (2013) performed genetic mapping (using SSR genotyping in 80 individuals of the < “Honey Blaze” × “D6090”> F1 progeny) to unveil the position of the dominant blood flesh (DBF) and the recessive blood flesh (bf) *loci* in the peach genome, proposing candidate genes for these functions (three genes of the dihydroflavonol-4-reductase). QTLs for total phenols, flavonoids, anthocyanins, and antioxidant activity were reported for the peach cross < “Venus” × “Big Top”> (Zeballos et al., 2016). Chagné et al. (2012) performed an UHPLC-DAD analysis of phenolic compounds in fruits from 170 F1 seedlings of the apple cross < “Royal Gala” × “Braeburn”>. In this analysis, the content of 17 compounds was associated through interval mapping (IM) to 79 QTLs, and seven of these *loci* were detected over two seasons. After the alignment to the apple reference genomic sequence, co-segregation analyses of candidate genes allowed the authors to indicate that leucoanthocyanidin reductase (LAR1) was a candidate for the control of (+)-catechin, (–)-epicatechin, and procyanidin oligomers biosynthesis, and hydroxycinnamate/quinate transferase (HCT/HQT) for the variation content of chlorogenic acid.

Although the cited data indicate that variation in the content of polyphenols is due, in part, to some *loci* in rosaceous fruits, the results from such strategies are not yet available for the Asian plum. In this species, QTL analysis based on the F1 progeny from the cross < “98.99” × “Angeleno”> indicated that, among other fruit quality traits, skin color (directly related to anthocyanin content) is associated with *loci* of LG3 and LG4, explaining together around a 70% of the trait phenotypic variance (Salazar et al., 2017). One such *locus* was mapped in a genomic position similar to the PsMYB10 gene sequence in chromosome 3 and was proposed for the early selection of plum individuals according to the predicted color (González et al., 2016b). In this current work, we performed the QTL analysis to detect *loci* correlated with phenolic compounds contents (PCC) in the < “98.99” × “Angeleno”> progeny, using genotypic data and linkage maps already available (Salazar et al., 2017, 2020) and phenotypic data obtained after two harvest seasons for PCC traits: total phenols, flavonoids, and procyanidins, along with antioxidant activity (DPPH and FRAP), and single anthocyanin compounds. This study constitutes the first step toward a genetic dissection of phenolic compounds in Asian plum.

## Materials and Methods

### Chemicals

Cyanidin-3-glucoside (C3G) and cyanidin-3-rutinoside (C3R) were purchased from Chengdu Biopurify Phytochemicals (China) and Extrasynthese (France), respectively. Merck (Santiago, Chile) supplied sodium hydroxide, sulfuric acid, high-performance liquid chromatography (HPLC)-grade acetonitrile, acetic acid, formic acid, and methanol. All reagents were of an analytical grade or higher. Polyethylene membranes of 0.22-μm pore size were acquired from EMD Millipore (Billerica, MA, USA). Gallic acid, catechin, dimethyl-amino-cinnamaldehyde (DMAC), FeSO_4_heptahydrate, trifluoroacetic acid (TFA), Trolox and Folin-Ciocalteu reagent were obtained from Sigma-Aldrich (Santiago, Chile).

### Plant Material and Fruit Samples

Two Asian plum cultivars (“98.99” and “Angeleno”) and 92 seedlings of the F1 progeny from their cross (< “98.99” × “Angeleno”>, hereafter “98Ang”) were planted at the Rinconada de Maipú Experimental Station (Santiago, Chile) in 2011. The maternal parent, “98.99,” is an early-medium maturing selection with red skin and yellow flesh of high organoleptic quality, while “Angeleno,” the paternal parent, has a late-medium maturity genotype with purple skin, yellow flesh, and excellent postharvest performance (Salazar et al., 2017, 2020). In this study, we considered fruits from 92 and 85 F1 seedlings of 98 Ang, harvested in seasons 2015–2016 (T1) and 2016–2017 (T2), respectively.

### Phenotypic Data

#### Phenological, Fruit Quality, and Color Traits

The samples employed in this work corresponded to the same fruits used for the studies of phenological and quality traits, reported in Salazar et al. (2017, 2020). The traits considered in this study are fruit development period (FDP), ripening time (RT), weight, coverage, maturity-related chlorophyll absorbance index (IAD) in skin and flesh, soluble solid content (SS), pH, and acidity. Fruit skin and flesh color were measured, using a CR-100 chroma-meter (Minolta, USA), calibrated with a standard white plate. Color measurements were recorded in CIE coordinates (L^*^: lightness, where black and white correspond to 0 and 100, respectively; a^*^: negative values for green and positive for red; b^*^: negative values for blue and positive for yellow). Chroma (C^*^ = [a^*2^ + b^*2^]^5^) and hue angle (H° = *arctan* [b^*^/a^*^]) were also measured with chroma-meter.

Harvest time was determined when the fruits reached IAD values between 1 and 1.4 units and a texture close to 40 N (Contador et al., 2016). Fruit quality traits phenotyping was carried out after 1 day at 25°C on 10 fruits per F1 seedling. Fruit replicates were labeled from 1 to 10. After fruit quality phenotyping, tissue samples of skin (“sk”) and flesh (“fl”) of three consecutively labeled fruits (i.e., fruits 1 to 3, 4–6, and 7–9) were pooled into three fruit replicates per tissue. In this way, on each season and for each tissue, we worked with three biological replicates corresponding to three different pools (the fruit sample and the data of the 10th fruit were not considered in this study). After phenotypic analysis, the samples were frozen in liquid nitrogen and stored at −80°C until their analysis.

#### Quantification of Phenolic Compound Families and Antioxidant Activity

The samples of skin (3 g) and flesh (10 g) were grounded in a laboratory mill (A 11 Basic, IKA, China) in liquid nitrogen (−196°C) until obtaining a homogeneous powder. Phenolic compound extraction was carried out by macerating samples in 10 ml of 80% methanol for 20 min on ice, followed by sonication for 20 min, and, finally, centrifugation at 8,000 g for 10 min at 4°C. Half of the obtained supernatant was concentrated in a 1:2 ratio to quantify phenolic compound families content, antioxidant activity, and single anthocyanins content. After concentration, the solutions were filtered (0.22 μm) and stored in amber vials at 4°C in darkness until analysis.

Quantifications of phenolic compound families content and antioxidant activity were performed in a 96-well plate format. Three technical replicates were considered for each fruit replicate. Calibration curves included 30 technical replicates per point. In total, on each season, 18 determinations were made per individual (two tissues x three [technical replicates/determination] x three [replicates/fruit]). An Infinite M200 Pro NanoQuant 96-well plate spectrophotometer was used (Tecan, CH). Mother and working solutions were kept at 4°C in darkness.

The total phenols (Phe) quantification was carried out with the Folin-Ciocalteu method adapted from Mubarak et al. (2012) for 15 μl samples. A calibration curve was constructed in the range of 100–600 μg/ml, using gallic acid as a reference. Absorbance was recorded at 765 nm, and the results were expressed as mg of gallic acid equivalents per 100 g of fresh fruit weight (mg GAE/100g FW).

The total flavonoid (Flv) content was determined, using the method adapted from Alvarez-Parrilla et al. (2011) to 15 μl samples. The calibration curve was in the range of 100–600 μg/ml of catechin in 80% methanol. Absorbance was read at 510 nm, and the results were expressed as mg of catechin equivalents per 100 g of fresh fruit weight (mg CAT/100-g FW).

Total procyanidins (Pca) determination was performed according to the DMACA method (Arnous et al., 2002), modified to 25 μl samples. For the calibration curve, concentrations between 5 and 50 μg/ml of catechin in 80% methanol were used. Absorbance was read at 640 nm, and the results were expressed as mg of catechin equivalents per 100 g of fresh fruit weight (mg CAT/100-g FW).

The antioxidant capacity of the extracts was estimated, using the ferric reducing antioxidant power (FRAP) and DPPH approaches to consider two different chemical mechanisms of radical reduction/scavenging by fruit tissue extracts. The FRAP method was carried out, following the protocol adapted from Yang et al. (2017) to a 2-μl sample/well. The calibration curve was constructed with FeSO4 heptahydrate solutions at concentrations ranging from 100 to 1,000 μg/ml in water. Absorbance at 593 nm was registered for each reaction, and the results were expressed in mmoles equivalent of Fe (II) per 100 g of fresh tissue (mmoleq Fe (II)/100-g FW). The stable radical DPPH elimination reaction method was adapted from Usenik et al. (2013) to 30 μl samples. The calibration curve was constructed with Trolox (D) solutions at concentrations ranging from 5 to 35 μg/ml in water. Absorbance at 520 nm was registered for each reaction, and the results were expressed in equivalent mmoles of Trolox per 100 g of fresh tissue (mmol TE/100-g FW).

#### Anthocyanin Identification by UHPLC-DAD-Orbitrap-MS

To identify the anthocyanins composition representative of the 98 Ang progeny, we prepared one mix of samples containing skin and flesh extracts from the parents and F1 individuals with contrasting color intensities. The analysis of the mixed sample was performed on an Acquity UHPLC system (Waters, Milford, MA, USA), coupled with an eLambda DAD (waters) and a High-Resolution Fourier Transform Orbitrap mass spectrometer, the/using the Exactive model (Thermo Scientific, Rodano, Italy), equipped with a HESI-II probe for ESI, operating in a positive mode and a collision cell (HCD). The operative conditions were as follows: spray voltage +4.0 kV; sheath gas flow-rate 55 (arbitrary units); auxiliary gas flow-rate 20 (arbitrary units); capillary temperature, 350°C; capillary voltage, 30 V; tube lens, 180 V; skimmer, 26 V; and heater temperature, 275°C. A BEH Shield C18 column (150 × 2.1 mm, 1.7 μm, waters) maintained at 50°C was used for the separation. The solution was centrifuged at 1,000 × g for 2 min, and 5 μL were injected into the UHPLC system. The flow rate was 0.5 ml/min, and the eluents were 0.01% trifluoroacetic acid in water (A) and acetonitrile (B). The UHPLC separation was achieved by the following linear elution gradient: 5–10% of B in 20 min, 10–20% B in 10 min, 20–45% B in 5 min, 45–90% B in 5 min, then 90–5% B in 1 min. The acquisition was made in the full-scan mode in the range (m/z)- 200–2,000 u, using an isolation window of ± 2 ppm. The AGC target, injection time, mass resolution, energy, and gas in the collision cell were 1 × 10^6^, 50 ms, 50 K, 30 and 80 V, N2, respectively. The MS data were processed, using Xcalibur software (Thermo Scientific). The peak identity was ascertained by evaluating the accurate mass, the fragments obtained in the collision cell, and the on-line UV spectra (200–600 nm).

#### Anthocyanin Quantification by the HPLC-DAD Method

After identifying the anthocyanins by UHPLC-DAD-Orbitrap-MS, we used cyanidin-3-glucoside (C3G) and cyanidin-3-rutinoside (C3R) as reference standards to determine the amount of anthocyanin in the extracts, using the HPLC-DAD technique, as reported by Peña-Neira et al. (2007).

To identify the peaks obtained in HPLC-DAD according to UHPLC-DAD-Orbitrap-MS results, we compared the profiles of the mixed sample (see section Anthocyanin Identification by UHPLC-DAD-Orbitrap-MS) obtained with both techniques and assumed a similar elution order of the different compounds among both chromatographic systems. The amounts of the anthocyanins that were not available as a reference standard were estimated, using the C3G calibration curve equation, and the results were adjusted by using a molecular weights ratio.

### Statistical Analysis of Phenotypic Data

Phenotypic data of PCC corresponded to spectrophotometric tests (phenolic compound families and antioxidant activities) and individual anthocyanins from HPLC. Previously available data for fruit quality traits of 98 Ang (Salazar et al., 2017, 2020) were also considered in this study. Matrices were generated in tab-delimited text format (“.txt”) for their analyses in R 3.6.1 software (R Core Team, 2018). All the considered traits, replicates, and individuals per season are summarized in Supplementary Table 1. Histograms were created, using the “hist” function included in the R software. Non-parametric Mann-Whitney *U*-test (“wilcox.test” function in R, one-tail, α = 0.05) was employed to compare progeny and phenotypic means of parents (assuming unequal sample sizes; Zimmerman, 1987) and thus provide an objective indication that the phenotypes changed their average value from one or both parents to the F1 generation. Correlation analyses among traits and within traits by tissue and season were performed, using the non-parametric Spearman method, considering a false positive rate of 0.001, using the packages “Performance Analytics” (Peterson and Carl, 2020), “corrplot” (Wei and Simko, 2017), and “Hmisc” (Harrell and Dupont, 2020).

For the broad-sense heritability determination, all traits were analyzed by ANOVA, which is included in the function “aov” of R. We considered the genotype (individual), year (seasons T1 and T2), and the fruit sample replicate as independent factors. We also included every non-ontologically related trait that significantly correlated with the trait being studied as a candidate covariate. The ANOVA was given by the general equation:

$$\begin{array}{l} y_{ijk}=u+G_{i}+Y_{j}+R_{k}+CovX+\;\varepsilon_{ijk} \end{array}$$

where *y*_*ijk*_ is the phenotypic value in the *i*th seedling in the *j*th year in the *k*^*h*^ fruit replicate, *u* is the overall average phenotype, *G*_*i*_ is the effect of the *i*th genotype (seedling), *Y*_*j*_ is the effect of the *j*th year, *R*_*k*_ is the effect of the *k*^*h*^ fruit replicate, *CovX* is the effect of a candidate covariate, and ε_*ijk*_ is the error term. The generic formula used in R was “aov(Trait~ Accesion + Year + RB),” adding the corresponding covariate. The broad-sense heritability (*H*^2^) of each trait was determined by dividing the genetic variance value of ANOVA by the total variance (Verdu et al., 2014), which was understood as the sum of the variances of each factor, including the residual variance. We expressed the obtained *H*^2^ value as a percentage.

### Linear Mixed-Effects Models and BLUP Estimation

For each PCC trait analysis, we estimated the phenotypic value of each F1 seedling, considering the effect of the environmental conditions and candidate covariates on PCC-traits for each replicated sample. The best linear unbiased predictors (BLUPs) were determined for every individual and trait through mixed linear models. The construction of these models began by including the fruit tissue replicate as a fixed variable and the “accession” (seedling) and “season” as random variables. We then iteratively included covariates that could increase the fit and improve the residual distributions. The determination of these covariates was based on the existence of significant correlations with the trait being studied. Each trait was analyzed, using mixed-effects linear models (MLM) with the R package “lme4” (Bates et al., 2015). The linear mixed models generated were adjusted, using the restricted maximum likelihood (REML) criterion, with the lowest value of the residual variance. The Akaike information criterion (AIC) was found to be the best fitted linear model. The functions “qqplot” and “qqnorm” were used to verify homoscedasticity and normality in the residual distribution of the generated models. To estimate the random effects due to the genotype and, thereby, obtain the phenotypic value for each F1 seedling, the BLUP value (Best Linear Unbiased Predictor) was extracted, using the “ranef” function. The BLUPs extracted from each model were used as phenotypic data in the QTL analysis.

### QTL Analysis

For the QTL analysis, we used the previously reported linkage maps of each parent (“98.99” and “Angeleno”) and the genotypic data of the 98 Ang progeny (Salazar et al., 2017). Genotype calling from GBS data was made by aligning tags with the peach genome V2.0 (Verde et al., 2017). SNP marker names corresponded to the peach genome position in which the Asian plum variant-containing tag had aligned (Salazar et al., 2017). Although a genomic sequence of *Prunus salicina* L. has been released recently (Liu et al., 2020), we did not update the GBS analysis since this manuscript had already been written. QTL analyses for PCC traits were performed in the MapQTL 6 software (Van Ooijen, 2009) in separate projects for parental genotypic and map data. The empirical LOD significance threshold (*p* < 0.05) was determined with the Permutation Test (PT), considering the genome-wide significance after 1,000 permutations, as implemented in the software. We used the Kruskal-Wallis non-parametric test and interval mapping (IM) to determine significant marker-trait correlations (LOD value higher than determined in PT). Subsequently, we determined marker cofactors with “automatic cofactor selection” to finally perform multiple QTL mapping analysis (MQM) to reduce the linkage-derived residual variance of QTLs and to detect possible QTL interactions. Linkage groups and QTLs were plotted, using MapChart 2.32 software (Voorrips, 2002).

## Results

### Detection and Quantification of Individual Anthocyanins by HPLC

After chromatographic analysis of the methanolic extracts corresponding to the progeny 98 Ang in two seasons, a total of 1,020 anthocyanin profiles were obtained (Supplementary Table 1 and Supplementary Figure 2). According to UHPLC DAD-Orbitrap-MS results, two main anthocyanins, cyanidin-3-glucoside (C3G) and cyanidin-3-rutinoside (C3R) were detected in all the analyzed samples (Supplementary Figure 1 and Table 1). The identity of C3G and C3R was subsequently confirmed by chromatography with authentic standards. In addition, smaller amounts of other anthocyanins were detected, which mass data suggested to be a cyanidin linked to different sugars and flavan-3-ol moieties. A summary of the tentatively identified anthocyanin compounds is shown in Table 1.

**Table 1:** Tentative identification of anthocyanins detected through HPLC-DAD and UHPLC-DAD-Orbitrap-MS.

**Average retention time (min)**	**λ max (nm)**	**m/z**	**Compound**	**Tentative identification**
4.18	281, 527	737.1700	D	Cyanidin-glucose-epigallocatechin
4.87	—	—	E	Not identified
5.61	—	—	F	Not identified
6.1	281, 517	883.2285	T	Cyanidin-glucose-rhamnose-epigallocatechin
6.8	285, 517	737.1704	L	Cyanidin-glucose-epigallocatechin
8.52	—	—	R	Not identified
8.86	277, 517	449.1067	C3G	Cyanidin-glucose
9.38	277, 517	595.1642	C3R	Cyanidin-rutinoside
11.45	281, 52	1025.2345	P	Cyanidin-glucose-epigallocatechin-epigallocatechin
11.91	—	1313.2992	M	Cyanidin-glucose-epigallocatechin-epigallocatechin-epigallocatechin
29.06	—	—	U	not identified

*The average retention time corresponds to the times recorded via HPLC-DAD for anthocyanins in both seasons and tissues. The λ max and m/z values correspond to the data obtained by UHPLC-DAD-Orbitrap-MS*.

### Phenolic Compounds Contents in 98Ang

Tables 2, 3 summarize the descriptive statistics obtained for PCC traits in the 98Ang progeny and their parents. It should be noted that PCC values are shown in the parents only for T1 since, in T2, there were no available samples for these varieties. As expected, the total of polyphenols resulted in higher amounts than flavonoids, procyanidins, and anthocyanins.

**Table 2:** Descriptive statistical parameters of phenolic compounds content in flesh.

**Trait**	**Season**	**Parent “98.99”**	**Parent “Angeleno”**	**98Ang progeny mean**	**98Ang progeny median**	**Shapiro-Wilk Test *p*-value**
Total phenols^a^	T1	119.14 ± 7.14	110.67 ± 18.26	126.33	116.08	0.00038
	T2	NA	NA	27.68	26.92	0.00033
Total flavonoids^b^	T1	12.99 ± 0.55	12.74 ± 0.56	32.10	20.07	0.00000064
	T2	NA	NA	15.29	14.64	0.000028
Total procyanidins^b^	T1	51.49 ± 4.20	32.45 ± 6.12	40.70	36.77	0.0000000027
	T2	NA	NA	8.56	7.57	0.000022
Total anthocyanins^c^	T1	1.49 ± 0.15	**2.38** **±** **0.50**	1.36	0.35	<2.2E-16
	T2	NA	NA	0.77	0.19	<2.2E-16
DPPH^d^	T1	1.2 ± 0.09	1.7 ± 0.30	1.14	1.06	0.0000000044
	T2	NA	NA	0.24	0.23	0.6791
FRAP^e^	T1	4.90 ± 1.9	4.46 ± 0.80	6.79	6.42	<2.2E-16
	T2	NA	NA	0.73	0.683	0.0003
C3G^c^	T1	**0.49** **±** **0.05**	**0.70** **±** **0.38**	0.55	0.12	<2.2E-16
	T2	NA	NA	0.33	0.05	<2.2E-16
C3R^c^	T1	**0.99** **±** **0.10**	**0.91** **±** **0.49**	0.76	0.20	<2.2E-16
	T2	NA	NA	0.32	0.06	<2.2E-16
Comp D^c^	T1	0.002 ± 0.001	0	0.0020	0.0010	<2.2E-16
	T2	NA	NA	0.001	0	<2.2E-16
Comp E^c^	T1	0.003 ± 0.001	0.002 ± 0.001	0.003	0.001	<2.2E-16
	T2	NA	NA	0.003	0.0003	<2.2E-16
Comp F^c^	T1	**0**	**0**	0.001	0	<2.2E-16
	T2	NA	NA	0.0004	0	<2.2E-16
Comp T^c^	T1	0.002 ± 0.0005	0.003 ± 0.0008	0.002	0.001	<2.2E-16
	T2	NA	NA	0.002	0	<2.2E-16
Comp L^c^	T1	**0**	**0**	0.001	0	<2.2E-16
	T2	NA	NA	0.002	0.0004	<2.2E-16
Comp R^c^	T1	0	**0.001** **±** **0.0007**	0.001	0.00006	<2.2E-16
	T2	NA	NA	0.001	0	<2.2E-16
Comp P^c^	T1	**0**	**0**	0.001	0	<2.2E-16
	T2	NA	NA	0	0	<2.2E-16
Comp M^c^	T1	0.007 ± 0.001	0.006 ± 0.004	0.009	0.0004	<2.2E-16
	T2	NA	NA	0.005	0.0004	<2.2E-16
Comp U^c^	T1	**0**	**0**	0.002	0	<2.2E-16
	T2	NA	NA	0.0003	0	<2.2E-16

*The parents mean (“98.99” and “Angeleno”) for each trait corresponds to the tissue replica (n = 3) from season 1, and its value is expressed together with its corresponding standard error. The means and medians of the 98Ang progeny for each trait correspond to the content of each individual in the population of each season (n = 92, T1; n = 80, T2). In the mean column of the parents, values highlighted in red represent significant differences with respect to the 98Ang progeny (see section Plant Material and Fruit Samples in Materials and Methods). The Shapiro-Wilk test p-value is highlighted in blue when higher than 0.05, indicating a normal distribution. Trait units are indicated by the following superscript symbols:*

a *, mg gallic acid eq./100-g FW;*

b *, mg catechineq/100-g FW;*

c *, mg C3G eq/100-g FW;*

d *, mmol Troloxeq/100-g FW;*

e *, mmol Fe(II) eq/100-g FW*.

**Table 3:** Descriptive statistical parameters of phenolic compounds content in skin.

**Trait**	**Season**	**Parent “98.99”**	**Parent “Angeleno”**	**98Ang progeny mean**	**98Ang progeny median**	**Shapiro-Wilk Test *p*-value**
Total phenols^a^	T1	496.87 ± 145.66	531.08 ± 95.71	449.66	408.05	0.00000012
	T2	NA	NA	125.42	120.9	0.00123
Total flavonoids^b^	T1	**136.21** **±** **28.46**	**127.79** **±** **4.11**	83.72	72.6	0.000000018
	T2	NA	NA	88.27	82.38	0.000008
Total procyanidins^b^	T1	**288.41** **±** **78.40**	**283.29** **±** **1.95**	167.95	144.35	1.1E-10
	T2	NA	NA	47.81	44.52	0.0000000034
Total anthocyanins^c^	T1	15.86 ± 3.91	**36.14** **±** **0.10**	18.03	13.97	<2.2E-16
	T2	NA	NA	13.39	10.23	<2.2E-16
DPPH^d^	T1	4.64 ± 0.13	**6.59** **±** **0.82**	3.74	3.62	0.0000089
	T2	NA	NA	1.05	1.05	0.0008211
FRAP^e^	T1	36.24 ± 10.75	**44.34** **±** **1.63**	32.91	31.21	0.00000052
	T2	NA	NA	4.65	4.33	0.0000087
C3G^c^	T1	3.06 ± 1.92	**6.16** **±** **6.15**	7.83	5.89	4.7E-12
	T2	NA	NA	5.83	4.33	<2.2E-16
C3R^c^	T1	7.35 ± 4.54	**5.66** **±** **5.64**	9.61	6.67	<2.2E-16
	T2	NA	NA	7.03	4.89	<2.2E-16
Comp D^c^	T1	0.007 ± 0.005	0.028 ± 0.015	0.024	0.013	<2.2E-16
	T2	NA	NA	0.007	0.001	<2.2E-16
Comp E^c^	T1	0.008 ± 0.004	0.022 ± 0.012	0.023	0.013	<2.2E-16
	T2	NA	NA	0.02	0.008	<2.2E-16
Comp F^c^	T1	0	0	0.002	0	<2.2E-16
	T2	NA	NA	0.001	0	<2.2E-16
Comp T^c^	T1	0	0.007 ± 0.004	0.004	0.002	<2.2E-16
	T2	NA	NA	0.017	0.005	<2.2E-16
Comp L^c^	T1	**0**	**0**	0.009	0	<2.2E-16
	T2	NA	NA	0.004	0.001	<2.2E-16
Comp R^c^	T1	0.007 ± 0.005	0.023 ± 0.012	0.016	0.01	<2.2E-16
	T2	NA	NA	0.013	0	<2.2E-16
Comp P^c^	T1	0	0.011 ± 0.006	0.014	0.006	<2.2E-16
	T2	NA	NA	0.006	0	<2.2E-16
Comp M^c^	T1	0.046 ± 0.03	0.095 ± 0.05	0.08	0.047	<2.2E-16
	T2	NA	NA	0.05	0.003	<2.2E-16
Comp U^c^	T1	0	0.009 ± 0.005	0.104	0.006	<2.2E-16
	T2	NA	NA	0.004	0	<2.2E-16

*Traits and unit specifications are the same as in Table 2. The Shapiro-Wilk test p-value is highlighted in blue when higher than 0.05, indicating a normal distribution*.

**Figure 1:**
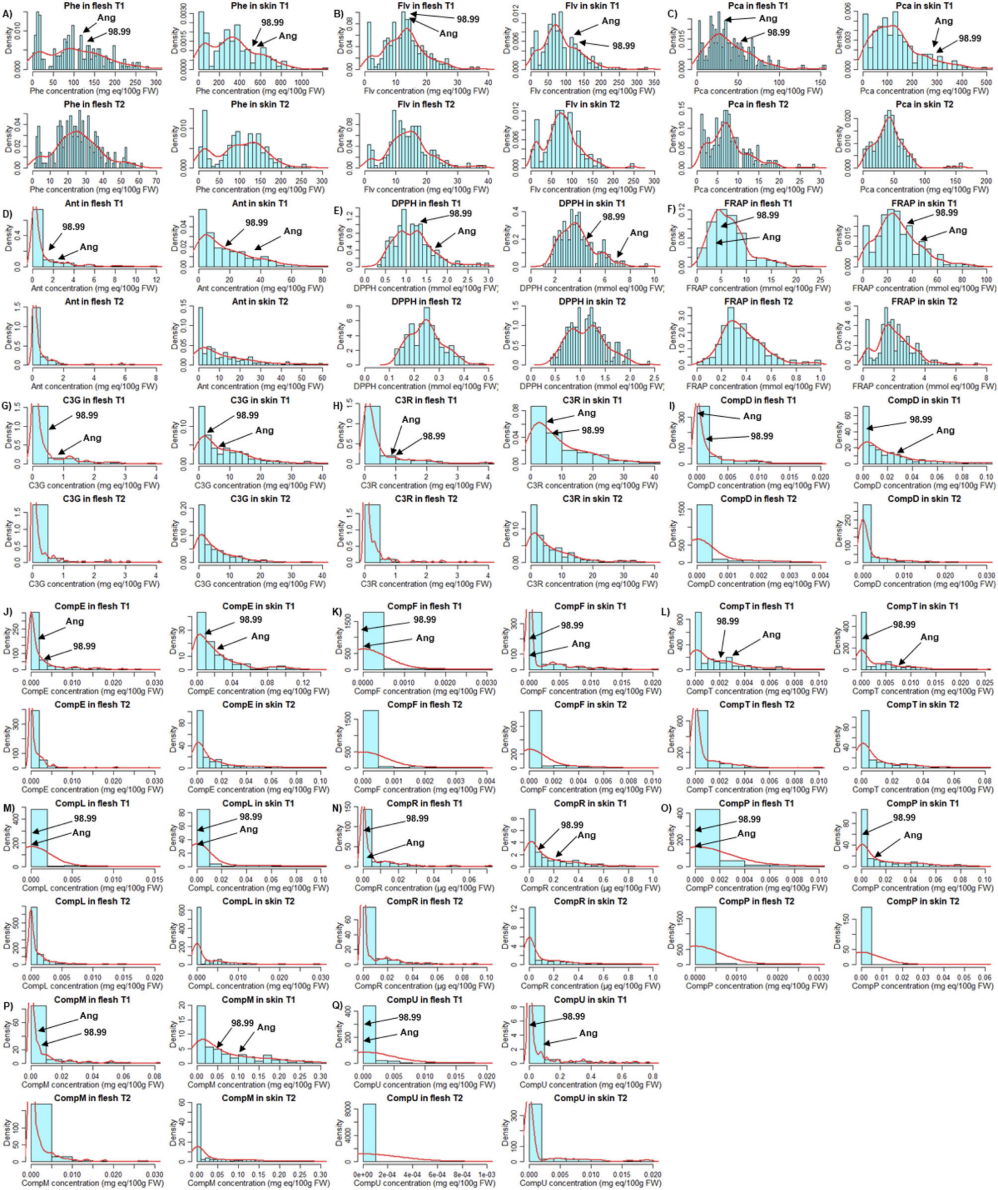
Data distribution of families of phenols and individual anthocyanins. Each histogram shows the distribution that follows the data obtained through spectrophotometric tests for phenol families and HPLC-DAD for the individual anthocyanins in plum samples. In each grouping, there are four distribution graphs corresponding to each tissue (skin and flesh) and each season (T1 and T2). The parents mean value (“98.99” and “Angeleno”) for each trait from season 1 is indicated with a black arrow. **(A)**: Phe, total phenols; **(B)** Flv, total flavonoids; **(C)** Pca, total procyanidins; **(D)** Ant, total anthocyanins; **(E)** DPPH, antioxidant activity test; **(F)** FRAP, antioxidant activity test; **(G)** C3G, cyanidin 3-glucoside; **(H)** C3R, cyanidin 3-rutinoside; **(I)** Comp. D; **(J)** Comp. E; **(K)** Comp. F; **(L)** Comp. T; **(M)** Comp. L; **(N)** Comp. R; **(O)** Comp. P; **(P)** Comp. M; and **(Q)** Comp. U. In each graph, the abscissa axis corresponds to the concentration in the units belonging to each test (see Materials and Methods).

In general, we observed that, for all types of compounds and quantified antioxidant activities (Tables 2, 3), the concentration values in skin (sk) exceeded at least two times that of the values observed in flesh (fl). Also, PCC and antioxidant activities detected in the T1 season were significantly higher than T2, indicating a strong environmental influence on the evaluated traits. Regarding the frequency histograms of 98Ang for each compound or antioxidant activity in each season (Figure 1), none of the evaluated traits showed a normal distribution, except DPPH antioxidant activity in mesocarp (fl) for the T2 season (Shapiro–Wilk test, Tables 2, 3).

To observe the potential additive effects transmitted from each parent to the F1 progeny, we carried out non-parametric comparisons (Mann–Whitney *U*-test; Zimmerman, 1987) of the different traits evaluated between each parent and the 98Ang progeny. Among the 34 traits evaluated (17 types of compounds quantified in two tissues, only in T1, 17 × 2 = 34; Tables 2, 3), we observed that, in 16, at least one of the parents showed a significantly different value in comparison to the population (eight in flesh and in skin; Tables 2, 3). Regarding the flesh content (fl), for C3G, the population mean (0.55 mg/100-g FW; Table 2) was in intermediate values to the mean of “98.99” and “Angeleno” (0.49 and 0.70 mg/100-g FW, respectively; Table 2), while, for C3R, the population mean (0.76 mg/100-g FW; Table 2) was lower than the mean of “98.99” and “Angeleno” (0.99 and 0.91 mg/100-g FW, respectively; Table 2), suggesting that *loci* having additive effects with a negative value for the concentration of C3R and C3G in flesh would be segregated in the progeny from both parents. For the anthocyanins compounds F, L, P, and U, the average obtained in both parents was 0, while, in 98Ang, the average value was 0.001-mg C3Geq/g FW, suggesting that it could be *loci* segregating with positive additive effects for the content of these compounds. In the case of phenol families in the skin, we observed that, for total flavonoids and total procyanidins, both contents of parents were significantly higher than the progeny averages (Table 3), indicating that they could be segregating *loci* with a negative additive effect. For the values of total anthocyanins, FRAP and DPPH, we observed that the parental “Angeleno” showed a significantly higher concentration than the progeny 98Ang average, as well an opposite effect to C3G and C3R was shown, suggesting that, probably, “Angeleno” and “98.99” contributed additive effects with positive and negative values, respectively.

### Correlations Between Phenolic Compound Contents

**Figure 2:**
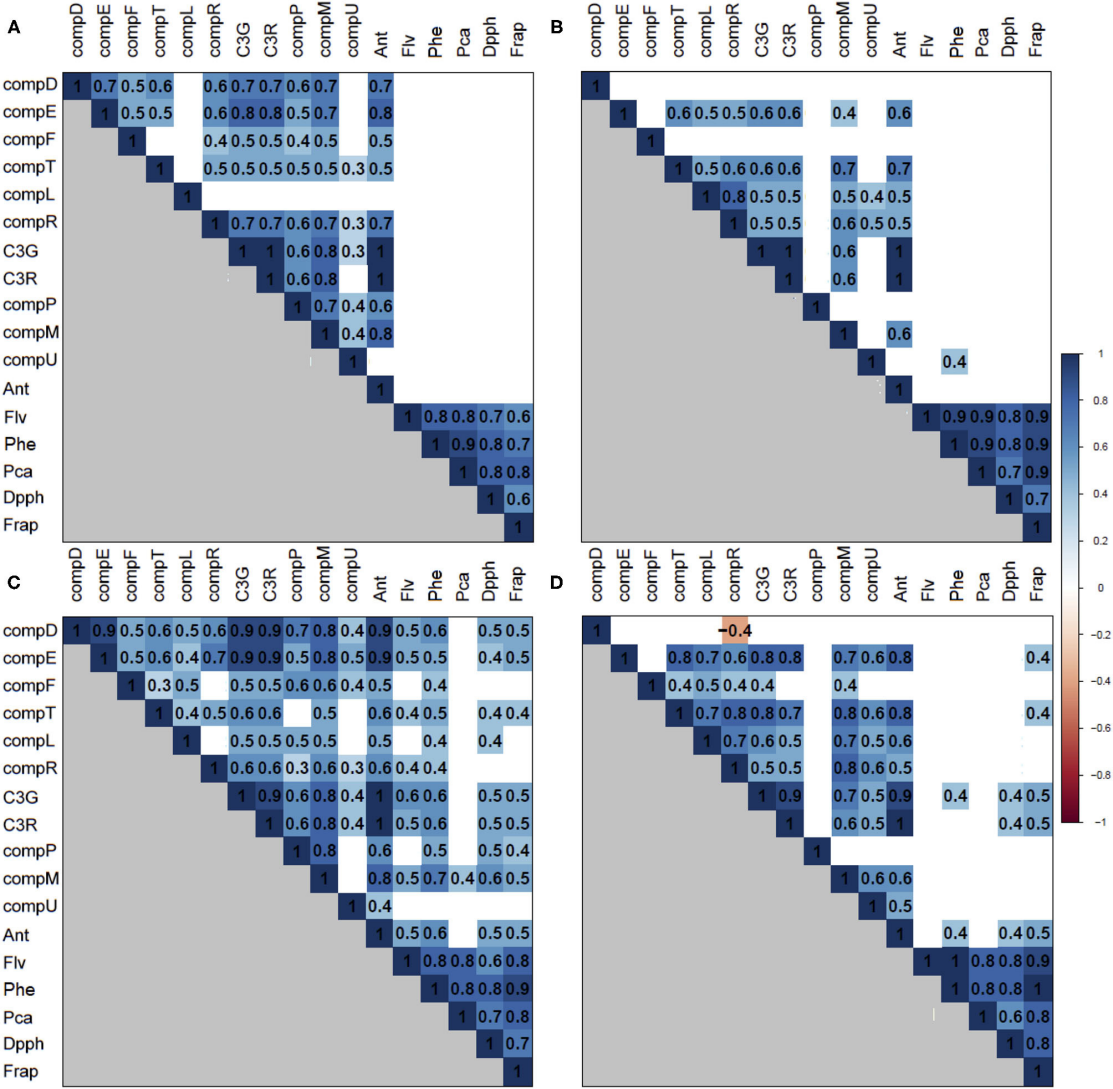
A correlation matrix between PCC traits for each season and tissue. The correlation matrix is based on a color gradient according to Spearman coefficients, where those with positive correlations close to 1 have a blue color and those with negative correlations close to −1 have a red color. The blank cells correspond to non-significant correlations (*p* > 0.001). **(A)** flesh, season T1; **(B)** flesh, season T2; **(C)** skin, season T1; and **(D)** skin, season T2.

To investigate whether there were correlations among PCC traits, per season and tissue, we used the non-parametric Spearman method (Figure 2). The highest and most significant correlations (*p* < 0.001) were found between phenol families and between C3G and C3R in both tissues and seasons.

**Figure 3:**
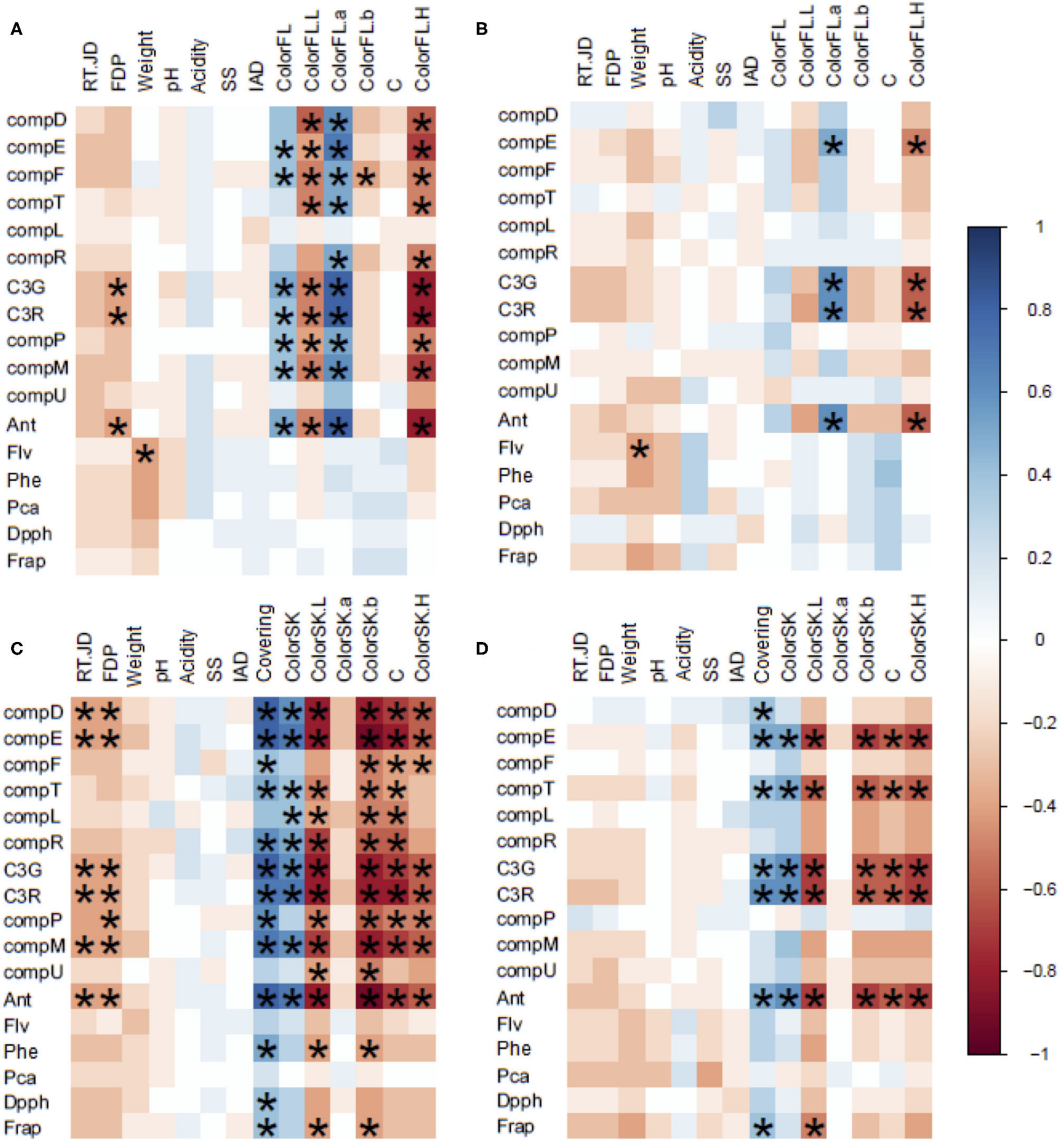
Matrix of correlations of phenotypic traits. The correlation matrix is based on a color gradient according to Spearman coefficients, where positive correlations close to 1 have a blue color, and negative correlations close to −1 have a red color. Significant correlations (*p* < 0.001) present an * in their box. **(A)** Correlation between phenotypic traits corresponding to flesh samples, T1; **(B)** correlation between phenotypic traits corresponding to flesh samples, T2; **(C)** correlation between phenotypic traits corresponding to skin samples, T1; and **(D)** correlation between phenotypic traits corresponding to skin samples, T2.

The correlations between flesh phenolic compound content in both seasons (Figures 3A,B) were higher than 0.7, except for DPPH and FRAP, and between total flavonoids and FRAP of season T1 (ρ = 0.6 in both). Furthermore, we observed that neither individual anthocyanins nor total anthocyanins correlated with the other phenolic compound families. The only anthocyanin that correlated with total phenols was the compound U in T2 (ρ = 0.4). These results suggest that, in the 98Ang progeny, the content of phenols, flavonoids, procyanidins, and antioxidant activities could be controlled by common genetic determinants but independently from anthocyanin regulation.

As for the flesh anthocyanins from season T1, the highest correlations (ρ > 0.7) were observed among C3G, C3R, and compounds D, E, R, and M. The correlation values among anthocyanin contents in T2 were lower than T1, where those with the highest correlations (ρ > 0.7) were C3G and C3R. Correlations between C3G and C3R were equal to 1 in both seasons. Compounds E and M correlated with a ρ = 0.8 in T1.

Regarding the skin, in both seasons, there were higher correlations (ρ > 0.8) than those observed in the flesh, as well as significant correlations (*p* < 0.001) between anthocyanins and the other families of phenols and antioxidant activities, although lower than 0.6. The correlations between phenolic compound families presented values ρ ≥ 0.8, with some exceptions, for example, between the DPPH and the rest of the compound families (ρ > 0.6) in both seasons. Among the T1 skin anthocyanins, those with the highest correlations (ρ = 0.8) were C3G, C3R, and compounds D, E, and M. In contrast, in T2 skin, C3G and compounds E, T, and M correlated strongly. The correlation value between C3G and C3R almost reached a maximum (ρ = 0.9). Finally, both anthocyanins in T1 correlated with compounds D and E with a value ρ = 0.9, and, in T2, they correlated with compounds E with a value ρ = 0.8.

In summary, these results showed that most of the relationships between PCC in this progeny are similar in the two seasons. Skin and flesh show diverse correlation patterns, indicating that metabolic pathways involved in phenolic compound biosynthesis are independently controlled.

The correlation for each tissue among seasons was examined to estimate stability of PCC traits across different seasons, and thus understanding the influence of environmental variations (Table 4 and Supplementary Figures 3, 4). Spearman correlation coefficients for each tissue are summarized in Table 4. We obtained significant (*p* < 0.05) but low correlation coefficients between data obtained in different seasons (ρ < 0.46). The highest correlations between seasons are found in flesh tissue and correspond to C3G, C3R, and total anthocyanins with values ρ ≥ 0.46. These data indicate moderate correlations between seasons for the analyzed single compounds and families, suggesting a medium and significant genetic effect, but a more substantial seasonal influence in the phenolic content in Asian plums of the 98Ang progeny.

**Table 4:** Inter-season phenotypic correlation coefficients (ρ) and broad-sense heritabilities (H^2^) of PCC-traits in 98Ang for skin and flesh tissues.

**Trait**	**Correlation (ρ)**		**Heritability (%)**	
	**Skin**	**Flesh**	**Skin**	**Flesh**
Total phenols	0.143^*^	0.137^*^	13.45	12.14
Total flavonoids	0.149^*^	0.268^**^	27.93	36.27
Total procyanidins	0.184^**^	0.194^**^	6.92	2.52
Total anthocyanins	0.406^***^	0.445^***^	29.27	22.12
C3G	0.393^***^	0.455^***^	31.39	23.82
C3R	0.442^***^	0.453^***^	32.04	17.27
Compund D	0.268^**^	0.17^**^	17.27	34.19
Compund E	0.368^***^	0.261^**^	25.23	20.23
Compund F	0.222^**^	0,057^NS^	17.37	7.83
Compund T	0.175^**^	0.293^***^	23.03	29.27
Compund L	0.024^NS^	−0.073^NS^	14.43	8.98
Compund R	0.17^**^	0.161^*^	3.74	29.91
Compund P	−0.124^*^	−0.085^NS^	18.21	3.01
Compund M	0.273^**^	0.16^*^	8.91	10.48
Compund U	0.269^**^	0.057^NS^	22.47	4.47
DPPH	−0.013^NS^	0.082^NS^	9.32	9.91
FRAP	0.238^**^	0.198^**^	2.36	4.00

*For correlation values, significance values are represented as follows: NS, non-significant*;

* *p < 0.05*;

** *p < 0.001*;

*** *p < 1 × 10^−6^*.

*For heritability values, only traits with resulting p-values higher than 0.05 were included*.

### Correlations Between Phenolic Compound Content and Fruit Quality Traits

To determine if fruit quality traits influence PCC traits in the 98Ang progeny, we performed correlation analyses between previously available fruit quality traits (Salazar et al., 2017, 2020) and phenolic content in each tissue and season through correlation analysis (Figure 3).

As in the intra-season correlations of anthocyanins, we detected more significant correlations between fruit quality traits in the skin than in the flesh. In both tissues, the greatest number of significant correlations were identified in the first season. High and inverse correlations between different anthocyanins (C3G, C3R, comp. E, and T) and fruit color parameters in both seasons and tissues were observed. Regarding the phenol families, these showed few correlations with the agronomic data for both tissues. Interestingly, in the flesh samples, we detected a negative correlation between total flavonoids and fruit weight (T1 and T2), suggesting that the flavonoid concentration is higher in seedlings with smaller fruits in this family. In skin samples of the T1 season, total phenols and FRAP were negatively correlated with color traits, and, together with DPPH, they correlated positively with the degree of color coverage in the fruit. With regard to the T2 season, only FRAP correlated negatively with color L and positively with coverage.

### Generation of Linear-Mixed Models and Estimates of BLUPs

The low inter-season correlations observed in Table 4 indicate a strong influence of environmental variables in PCC traits. For this reason, we decided to estimate predictors of additive genetic effects for the 98Ang progeny. Supplementary Tables 3, 4 show the variables included in the models for each trait. In Supplementary Figures 5–8, the distribution and the normality of the residuals (Q-Q plot) are shown as a model evaluation criterion.

Broad-sense heritability for the analyzed traits in the flesh reached a maximum of 36.3% in the case of total flavonoid content. On the other hand, the lowest heritability (2.5%) corresponded to the total procyanidins (Table 4).

In the skin, the highest heritability value corresponded (32.04%) to the C3R, while the lowest (2.4%) corresponded to FRAP antioxidant activity (Table 4).

The low heritability values observed in both tissues confirm a significant influence of environmental variables on the phenolic compound traits. Such an effect is higher in the skin than in the flesh, probably due to the former tissue is subjected to slight intraplant or intra-fruit environmental variations. In contrast, the moderate values in the traits Flv-fl, C3G-sk, C3R-sk, and compD-fl indicate that significant genetic components could be found in the QTL analysis.

### QTL Analysis

The corrected F1-individual phenotypic values (BLUPs) for each trait were used to analyze QTLs, jointly with the genotypic data and the genetic maps of each parent of 98Ang, generated by Salazar et al. (2017). QTLs associated with phenolic compounds, mainly present in the skin, were detected in all linkage groups, except for linkage groups (LG) 2 and 6. The main informative parameters of the QTL analysis are described in Supplementary Table 5.

**Figure 4:**
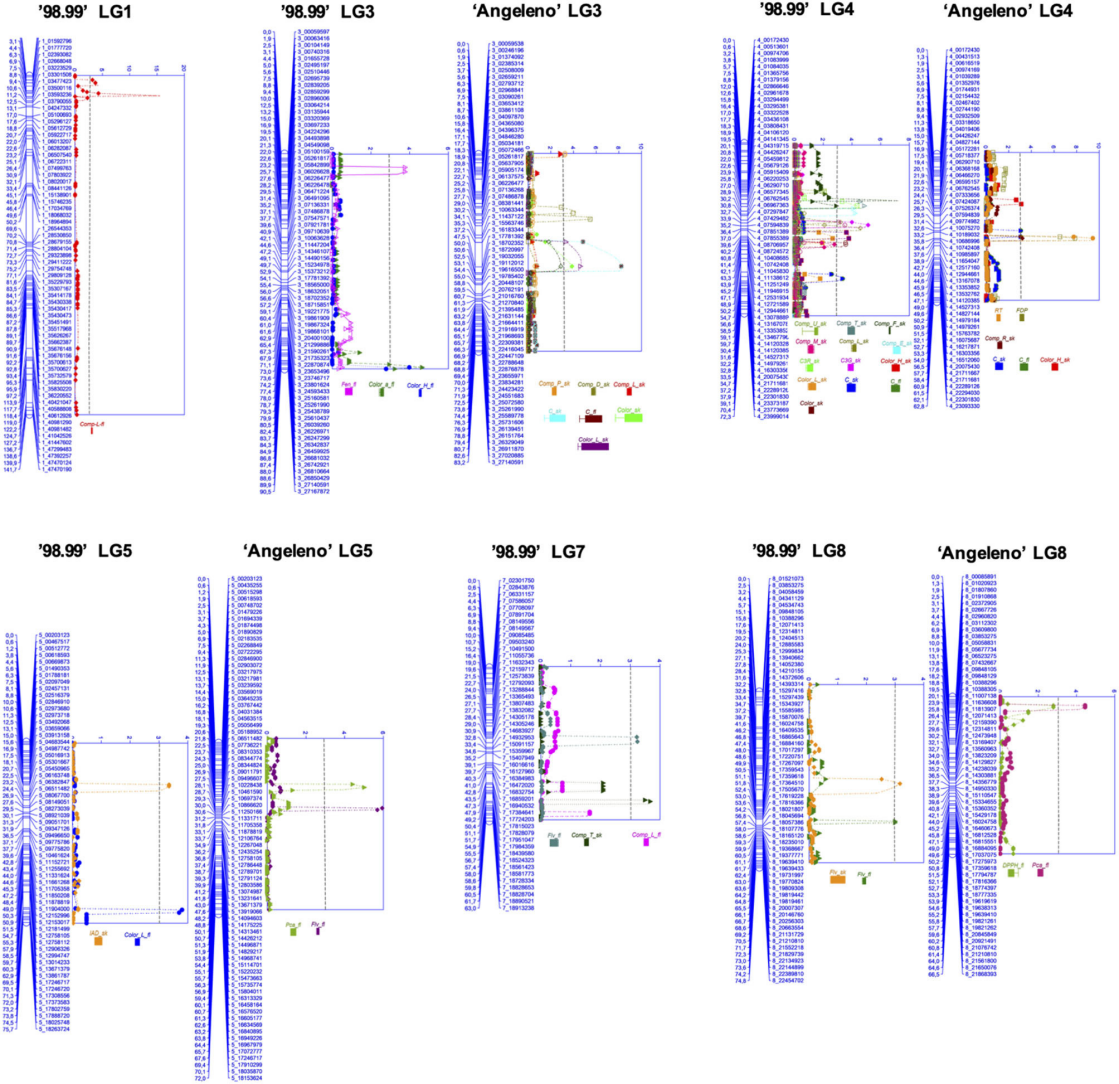
Multiple QTL model results for PCC traits in “98.99” and “Angeleno”. Each QTL is assigned with its corresponding trait, using the same names used in Supplementary Table 5. The abbreviation “fl” next to each name indicates that it comes from flesh tissue. On the left side of LG, the position (cM) of each marker is indicated and, on the right, is the marker associated with that position. The graph to the right indicates the LOD values of each QTL identified by color. All QTLs detected exceeded the limit LOD value reported in the QTL analysis of each trait evaluated, indicated in Supplementary Table 5 (permutation test value). A dotted gray line indicates LOD level 3 to indicate a reference significance level, common to all found QTLs.

In the genetic map of “98.99,” 13 QTLs were detected, most of them corresponding to single anthocyanin content in skin, concentrating mainly on LG4 (Figure 4). QTLs from this parent explained a range between 13.1 and 26.7% of trait phenotypic variation, corresponding to the total content of flavonoids (flesh, LG8) and C3G (skin, LG4), respectively. In the parent “Angeleno,” there were eight QTLs associated with phenolic compounds, distributed mainly between the LG3 and LG5. The phenotypic variation explained by each QTL (to the corresponding trait) was found in the range between 12.4 and 27.1%, belonging to the QTLs compound L (skin, LG3) and total flavonoids (flesh, LG5), respectively.

The LOD value of QTLs segregating from “98.99” varied between 2.77 and 5.12, while QTLs from “Angeleno” varied in a range between 2.79 and 5.69. In both parents, the reported LOD value was higher than the LOD value limit determined by the Permutation Test (PT) in analyzing QTLs for each trait evaluated. The minimum LOD values ranged from 2.3 to 2.8, depending on the trait (Supplementary Table 5). Despite this, the detected QTLs in both parents, for the compound U, did not have a significant LOD value (2.07 and 0.96 for “98.99” and “Angeleno,” respectively), and therefore, were discarded as candidate QTLs.

Linkage groups of each parent, together with their QTLs, are presented in Figure 4. In LG8 of “Angeleno,” the QTLs of total procyanidins and DPPH in the flesh (Figure 4 and Supplementary Table 5) shared a similar position (SNP 8_02960820, 4.356 cM), suggesting that procyanidins would mainly be the compounds responsible for the antioxidant capacity in the flesh of the fruit. Another interesting QTL cluster was found, as mentioned above, in LG4 of “98.99,” in which the most extensive QTL cluster associated with individual anthocyanins was detected, including C3G and C3R (Figure 4), where seven QTLs were positioned in a small range within this linkage group (from 20.14 to 35.16 cM), while the QTL of compound M was located at 42.05 cM. All of these QTLs were found in the skin, co-localizing with QTLs associated with color traits, suggesting the complex network of participation of various genes in the pigmentation process of the skin of the fruit through the synthesis and accumulation of anthocyanins, such as C3G and C3R. Identifying candidate genes related to the regulation, transport, and biosynthesis of phenolic compounds in those QTLs is an essential step toward a better understanding of the production of these secondary metabolites and thus a tool for the future development of genetic improvement programs in plum trees. In addition, the most significant skin color QTL (C^*^) was located in the LG3, which reached a LOD value of 8.22 in the “Angeleno” parent, collocating with the SNP 3_17781392 (Supplementary Table 5), which is located near the gene coding for the MYB10 transcription factor previously associated with skin color in Asian plum (González et al., 2016a,b). Similarly, different phenolic compounds as well as skin color co-localized in the LGs 3 and 4. Therefore, the implication of these chromosomes on skin color variations, phenolic compounds, and even the ripening time or fruit development period is proved. Thus, these findings open up the possibility to molecular-assisted selection for these traits.

## Discussion

### Phenolic Compound Content in 98Ang

In this study, we determined the content of phenolic compound families, single anthocyanins, and antioxidant activity in fruits of the Asian plum cultivars “98.99,” “Angeleno,” and the progeny from their cross (98Ang). The procedures for organic extraction and analyses were chosen to satisfy the need to process a high sample quantity quickly and with good precision. For total phenols, the progeny mean values ranged from 27.68 to 126.33 and 125.42 to 449.66 mg GAE/100 g FW in flesh and skin tissues, respectively. According to Diaz-Mula et al. (2009) and Gil et al. (2002), the total phenolic content determined in four cultivars for each work ranged from 38 to 124 and 163 to 521 mg GAE/100-g FW in flesh and skin, respectively, indicating that the estimation of total phenols in our study was in the range reported in the literature (Fanning et al., 2014), although slightly lower. In Gil et al. (2002), two black-skinned (“Angeleno” and “Black Beaut,” 332- and 318-mg GAE/100-g FW, respectively), and two red-skinned Asian plum cultivars were included (“Red Beaut” and “Santa Rosa,” 163- and 166-mg GAE/100-g FW, respectively). Total phenolic content in the skin of the black varieties was two times that of the red ones, indicating a large variation in this trait according to external fruit color. Moreover, Diaz-Mula et al. (2009) reported high variability among black-skinned Asian plum cultivars (“Angeleno,” “Black Amber,” “Larry Ann,” and “Black Diamond”), which ranged from 270-mg GAE/100-g FW in “Black Diamond” to 521-mg GAE/100-g FW in “Black Amber.” The total phenolic content in cultivar “Angeleno” reported in the present study (531.08-mg GAE/100-g FW in the skin and 110.86-mg GAE/100-g FW in the flesh, both in the season T1), in Gil et al. (2002) (332-mg GAE/100-g FW in the skin and 41-mg GAE/100-g FW in the flesh), and Diaz-Mula et al. (2009) (360-mg GAE/100-g FW in the skin and 100-mg GAE/100-g FW in the flesh) shows high dispersion among studies. Environmental and analytic differences among studies can explain this variation, and further studies, including more cultivars of different flesh and skin colors sampled in different environments, are necessary to characterize the variation of this trait in Asian plum.

The results obtained in this study were also in agreement with Speisky's et al. (2012), where the total phenolic content and ORAC antioxidant activity of “Black Amber” Asian plum cultivar (150-mg GAE/100-g FW in flesh) were ranked together with other widely consumed fruits and positioned them in the eighth place after custard apples, blueberries, blackberries, walnuts, and three edible Patagonian fruit species. The study showed Asian plums as having one of the highest phenolic content among rosaceous fruits, without considering blackberries. In the 98Ang progeny, the most represented phenolic compound family was the total procyanidins, with progeny mean values of 8.56-mg catechineq/100-g FW in T2 and 40.70-mg catechineq/100-g FW in T1 in flesh tissue, and 47.81-mg catechineq/100-g FW in T2 and 167.45-mg catechineq/100-g FW in T1 in skin tissue, in agreement with Ogah et al. (2014) where a range between 66.2- and 183.7-mg catechineq/100-g FW is reported for peeled plum. As for antioxidant activity, our DPPH results in flesh (0.24 and 1.14-mmol TE/100-g FW in flesh and skin tissues, respectively), were slightly lower than in Venter et al.'s (2014), where the values between 1.14 and 1.77 mmol TE/100-g FW were observed in a comparison of flesh from south African plum cultivars. Regarding FRAP, our value range in flesh [0.73- and 6.98- mmol Fe(II)/100-g FW in flesh and skin tissues, respectively], was higher than the data of Venter et al. (2014) [0.93–1.52-mmolFe(II)/100-g FW]. Regarding this point in our data, we observed that flesh DPPH values were higher in “Angeleno” than in “98.99” (1.7- and 1.2- mmol TE/100-g FW, respectively), while for FRAP data in the same tissue, the opposite is observed (4.46- and 4.90- mmol TE/100-g FW for “Angeleno” and “98.99,” respectively), which can be explained for the high dispersion obtained in FRAP assays. This high standard deviation observed among FRAP replicates indicates that this technique could be less adequate for comparing plum samples with similar phenolic compound content.

### The Environmental Influence on Phenolic Compounds

Improving the quality of fruits in phenolic components is a challenge in fruit-breeding programs because these traits are quantitatively inherited; the content of such metabolites is controlled by several genes, in addition to being strongly influenced by the environment, such as a pathogen attack, exposure to high ultraviolet radiation, and the presence of pollinators (Howell, 1974; Beckman, 2000; Jeandet et al., 2002; Jakubska et al., 2005; Zhang et al., 2016). Therefore, to separate the heritable components from the non-heritable ones, it is essential to establish appropriate selection methods in breeding programs (Oliveira et al., 2014). The observed environmental effect in the PCC in different Rosaceae fruit species has been reported in several studies. Abdelghafar et al. (2020) found 14 QTLs associated with phenolic compounds in the peach genome, of which only one QTL was stable through the 2 years analyzed. Similar results were obtained by Chagné et al. (2012), who detected 79 QTLs for 17 phenolic compounds present in the apple genome, with only seven groups of stable QTLs in 2 years of evaluation, representing seven classes of phenolic compounds. Finally, although Verdu et al. (2014) obtained high heritability values in apple extracts (0.57–0.98), of the 171 QTLs detected, a few of them were significant across the 3 years of evaluation, representing only four classes of phenolic compounds. In this work, the 98Ang F1 progeny showed remarkable differences in PCC in both seasons, with the first season being higher than the second season (Figure 1). The Spearman correlations between seasons (Table 4) were low (ρ ≥ 0.46) for both tissues analyzed, a behavior that was also observed by analysis of variance. Thus, the factor “season” had a significant impact on the parameters for all the examined traits and was subsequently included as a random-effect variable in the generated linear-mixed models. The observed differences can be explained due to eventually different environmental conditions among seasons. Climatic variables, such as UV radiation, are important *stimuli* that modify the phenolic compound content in fruits (Ogah et al., 2014). We did not notice any contrasting differences between years in temperature, UV radiation nor precipitation throughout the fruit development period after checking the available climatic bulletin with monthly data (Dirección Meteorológica de Chile, 2021). More precise studies could be useful to associate slight changes to the dramatic observed phenotypic variation.

Despite the environmental influence over PCC traits, additive effects transmitted from each parent to the progeny (Tables 2, 3) are suggested by comparing the PCC of parents and the population. Together with the broad sense heritability values (36.3% for Flv, Table 4), these results indicate the presence of genetic components with significant effects, as detected in the QTLs analysis of Oliveira et al. (2014), following a similar analysis of variance in root traits studies in cassava. For this reason, we could detect significant genetic effects that allowed to estimate BLUPs for each seedling of the 98Ang progeny from linear-mixed models of each trait (Supplementary Tables 3, 4).

### Relations Between Phenolic Compounds and Fruit Quality Traits

Accumulation of phenolic compounds in fruits is closely related to their ripening parameters (Andreotti et al., 2008). In this way, slight environmental and positional differences within the plant could lead to variations in the studied traits. Hence, PCC data were analyzed together with a fruit quality phenotypes dataset (Salazar et al., 2017, 2020) to consider the variations among fruit tissue replicates in ripening-related fruit quality traits (SSC, IAD index, and firmness) and to obtain corrected phenotypes and thus more precise QTL results.

As expected, we found that anthocyanins C3G and C3R are associated with the coverage of plum skin color. Their QTLs were located in the same linkage group at “98.99” (LG4: 33.9 cM for C3G and 25.8 cM for C3R), similar to Salazar et al. (2017), where QTLs for skin color (SKC: 25.0–52.0 cM) and over-color (OVC: 25.9–59.6 cM) traits were found at “98.99,” within the LG4. The QTL cluster found in LG4 of “98.99” is located in a region containing at least two candidate genes in the peach genome V.2.0, with sequence similarity for transcription factors with possible participation in the regulation of phenolic compounds biosynthesis, such as Prupe.4G126900.1 (with sequence homology to R2R3-MYB transcription factor from mulberry; Li et al., 2020), Prupe.4G134200.1 (with a sequence homology to bHLH52 DNA binding protein from a grapevine; Hichri et al., 2010). To confirm the involvement of these genes in the Asian plum anthocyanin content variation, fine-mapping of this QTL is needed to narrow this region (with the phenotypic and genotypic analyses of F1 recombinants in this region), in addition to sequence variant co-segregation analyses in this genomic region of “98.99.” However, these data are not available to date, and further studies are needed. Although the presence of these genes in the region indicates the functional validity of the found QTL, the presence of a causal mutation needs additional sources of evidence to be proved.

Over-color QTLs have also been found in other species and varieties of *Prunus* in different linkage groups, such as the peach (*Prunus persica* [L.] Batsch) in LGs 3, 4, 5, 6, and 7 (Eduardo et al., 2011; Hernández Mora et al., 2017). In the cherry (*Prunus avium* L.), QTLs were found for red skin color in linkage groups 3, 6, and 7 (Sooriyapathirana et al., 2010) and in the apricot (*Prunus armeniaca* L.), QTLs were identified in LGs 2, 3, and 6 (Ruiz et al., 2010; Socquet-Juglard et al., 2013; García-Gómez et al., 2019). A candidate gene has been found in LG3, which codes for the MYB10 transcription factor, induced by exposure to sunlight, regulating anthocyanin biosynthesis, generating red coloration in fruits (Takos et al., 2006). Anthocyanins C3G and C3R are responsible for the red-violet pigmentation in the skin (Gao and Mazza, 1995; Mozeti et al., 2002; Fanali et al., 2011; Mikulic-Petkovsek et al., 2016), whose biosynthesis is regulated by MYB10 and other still unknown genetic components present, for example, in the QTLs of LG4 from “98.99.”

Unexpectedly, although anthocyanin content in 98Ang significantly correlated with color traits in the skin (Figure 3), we did not find any QTL for anthocyanin content co-localizing with color-related QTLs in the LG3 of “Angeleno” (except for compounds L, P, and U; Figure 4 and Supplementary Table 5). In this work and the studies in the *Prunus* species cited above, QTLs located in the LG3 are related to fruit skin color: blush or over-color; however, none of them directly analyze the anthocyanin content in stone fruits. In the peach, Abdelghafar et al. (2020) and Font iForcada et al. (2019) found QTLs associated with anthocyanin compounds through linkage-based QTL mapping and GWAS, respectively. No QTLs were found in LG3 for anthocyanin content but only in LG5 in Abdelghafar et al. (2020) and LGs 1, 2, and 4 in Font iForcada et al. (2019). These data suggest that an unknown interaction could be causing the observed gap between color-anthocyanin correlations and detected QTLs.

### Identification of QTLs Associated With Phenolic Compounds

Identifying QTLs associated with phenolic compounds is an essential step for the future generation of fruit varieties with added value in their antioxidant function. Furthermore, the identification of QTLs allows for a better understanding of the biosynthesis pathways of these compounds, where various metabolic steps related to their synthesis, transport, and storage sites remain unknown (Zhao and Dixon, 2010; Verdu et al., 2014; Xu et al., 2015; Zhao, 2015). In the 98Ang F1 population, the traits associated with phenolic compounds showed significant differences between the evaluated seasons, which indicated a strong environmental influence. Hence, the broad sense heritability was moderate yet significant (2.36–32.04%), showing that it is possible to determine *loci* controlling these traits. There are few works reporting heritability values for stone fruits. Recently, Karaat and Serçe (2020) have determined moderate broad-sense heritability values for total phenolic compounds and antioxidant activity in apricot progenies (14 and 43%, respectively), although higher values have been found in raspberry (narrow-sense: 48–54%; Connor et al., 2005) and blueberry progenies (38–56%; Connor et al., 2002). These data indicate that, in the mentioned *Prunus* species, heritabilities are lower than in other Rosaceae and Ericaceae fruits. However, heritability studies in raspberry and blueberry considered data from several progenies, in contrast to the results reported in this work, which are based only on 98Ang progeny. Moreover, to give a deeper characterization of environmental and genetic variance components involved in phenolic compound content, additional seasons should be analyzed in further studies.

The use of mixed-linear models to obtain the genetic components of trait variance is widely used in breeding programs for various plant species (Salazar et al., 2020), allowing a more precise approach in the QTL analysis by integrating influence of environmental factors on the evaluated traits. Consequently, it was possible to find QTLs related to phenolic compounds from “98.99” in LG 1, 3, 4, 7, and 8, most of which were concentrated in LG4 and LG7, while in “Angeleno,” QTLs were identified only in the LGs 3, 4, 5, and 8. A similar distribution of phenolic QTLs was also found in other stone fruit species, although in different positions (Abidi, 2012; Zeballos et al., 2016; Abdelghafar et al., 2020), which indicated the complex and organized network of genetic components specialized in the production, accumulation, and transport of these plant secondary metabolites.

For families of phenolic compounds, a QTL for total phenols in the skin was detected in the top of LG3; four different QTLs for total flavonoids were found in the skin and flesh in LG5, LG7, and LG8. For total procyanidins content in the flesh, QTLs in LG5 and LG8 were found. Similar results were reported by Zeballos et al. (2016) with an F1 population of nectarines (*Prunus persica* [L.] Batsch) derived from the cross between “Venus” and “Big Top,” where a QTL associated with total phenols in both parents was found in LG2 and 5 QTLs in the linkage groups 2, 3, 4, and 7 associated with the accumulation of flavonoids. In this regard, Abdelghafar et al. (2020) used a peach F2 progeny (F2 between “Zin Dai” and “Crimson Lady”) to detect three QTLs associated with flavonoid content and were located in LG7 and LG5. Together, these results suggest a conserved region in the *Prunus* species to control flavonoid content in LG7 and LG5. As for the QTL found in “Angeleno” LG5, controlling total procyanidin content in flesh (19.35–21.56 cM), we located a candidate gene in peach genome (Prupe.5G061400.1; InterPro term IPR001471) coding for an AP2/ERF domain protein. The function and regulation networks of these transcription factors have been associated to the control of the abiotic stress response in apple by regulating the biosynthesis of procyanidins (Zhang et al., 2018). Thus, we suppose that the functional role of this QTL in the synthesis of procyanidins is based on stress-induced regulatory mechanisms in the fruits.

Regarding antioxidant activity, specifically in the flesh, a single QTL in LG 8 was identified, which is in the same linkage group reported by Zeballos et al. (2016), who also found another QTL related to the antioxidant capacity in LG4. Abdelghafar et al. (2020) detected two QTLs associated with antioxidant activity in LG1 and 5. However, none of the QTLs identified by them were stable for the two seasons analyzed (2013 and 2014), suggesting that these traits were strongly affected by climatic events or the management of these cultivars in each season. In turn, presently, the QTL for flesh DPPH antioxidant activity is grouped with the QTL of total procyanidins, suggesting that these phenols could be responsible for the antioxidant activity in the flesh of the plum.

Regarding single anthocyanins, QTLs were identified in linkage groups 1, 3, 4, and 7, concentrating mainly on LG3 and LG4, where the main anthocyanins, C3G and C3R, were found in the latter QTL. These results differ from previous reports (Zeballos et al., 2016; Abdelghafar et al., 2020), which detected QTLs for total anthocyanins in LG5 and LG7, respectively. In this work, no QTL was detected for total anthocyanins. However, knowing that C3G and C3R are the most abundant anthocyanins in the 98Ang progeny, it follows that this trait would have a QTL in the same position (LG4), so the differences reported could be due to the occurrence of different types of anthocyanins present in *Prunus persica (L.)* Batsch and *Prunus salicina* L.

In all of the analyses of QTLs associated with phenolic compounds, the associated *loci* are dispersed throughout the *Prunus salicina* L. genome. These *loci* are mostly forming clusters, suggesting that, for several phenolic compounds, there are common regulatory elements that control their regulation/biosynthesis, thus confirming the complexity in the production of these secondary metabolites (Naczk and Shahidi, 2004; Takos et al., 2006).

In this work, we obtained results that indicate a high complexity in the genetic dissection of PCC traits in Asian plum due to a strong environmental influence on these traits. Despite these difficulties, we could define regions of the Asian plum genome that control the phenolic profile in the fruit. These QTLs explained between 12.4 and 27.1% of trait variance in a group of traits highly influenced by the environment. We also found QTLs in LG3 from “Angeleno” for external fruit color with a PEV of 32% (C^*^ parameter), which could be important for assisted selection regarding fruit color. The regions found in this study provide a first step in unveiling the mechanisms involved in the regulation, biosynthesis, or transport pathways of phenolic compounds with antioxidant capacity. However, for a more precise dissection of the PCC traits, it will be useful for further studies to characterize the environmental sources of their variation, for example, including additional seasons or locations with available climatic data; also, to consider more crosses could possibly show the involvement of new *loci*-carrying variants with an impact on the phenotypic value for PCC traits, allowing to characterize the genetic architecture of phytochemical content in the Asian plum. Finally, after a transferability validation, these QTLs and linked markers could be considered as selection tools for managing phenolic compound content in new cultivars.

## Data Availability Statement

The original contributions presented in the study are included in the article/Supplementary Material, further inquiries can be directed to the corresponding author/s.

## Author Contributions

DV-S performed data analysis and wrote the manuscript. IP developed the concept of the work, applied for funding, coordinated experimental work, and supervised manuscript writing. RI developed the crosses and F1 populations and maintained them on the field in good condition for this study. JS coordinated harvest, quality analysis, and fruit sampling. DV-S, AS-G, CS-A, ÁP-N, and HM participated in the quantification and analyses of the compounds. CG performed MS compound identification. RI, PJ-M, BB, MG, and JS participated in editing and preparation of the final version of the manuscript. All authors contributed to the article and approved the submitted version.

## Conflict of Interest

The authors declare that the research was conducted in the absence of any commercial or financial relationships that could be construed as a potential conflict of interest.
