# Supplementary material for: Detection of Quantitative Trait Loci Controlling the Content of Phenolic Compounds in an Asian Plum (Prunus salicina L.) F1 Population
Source: Front Plant Sci. 2021 Jul 9;12:679059. doi: 10.3389/fpls.2021.679059 (PMC8299277; doi:10.3389/fpls.2021.679059)
Supplement: Supplementary file 1 [file Presentation_1.PDF]

Supplementary Material

1 Supplementary Figures

A)

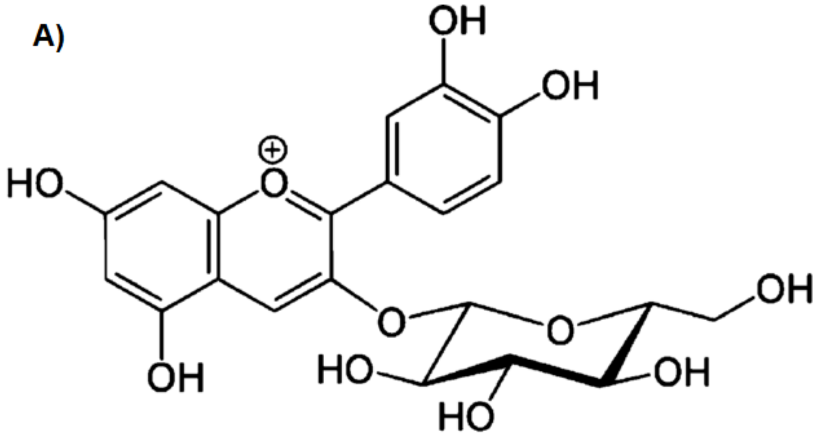

B)

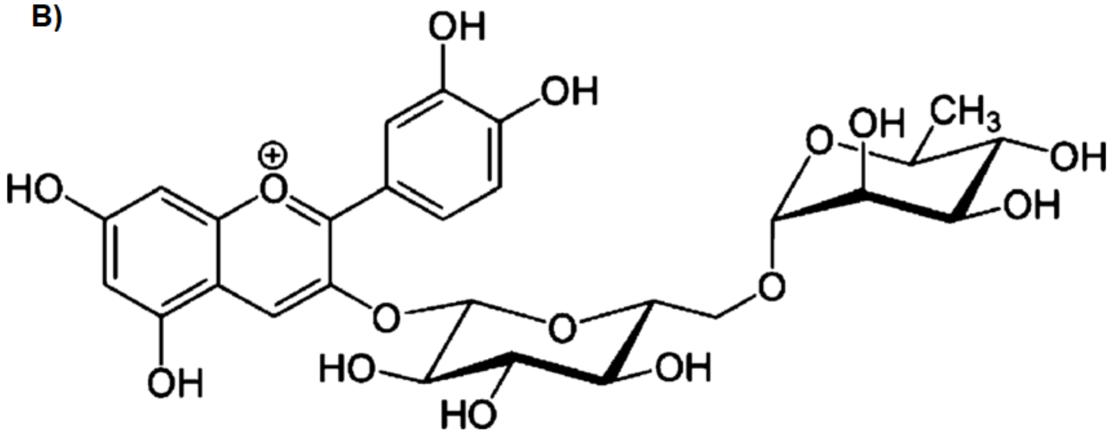

Supplementary Figure 1. Chemical structure of principal individual anthocyanins detected. A. Cyanidin-3-Glucoside (C3G); B. Cyanidin-3-Rutinoside (C3R). Image taken and modified from Kamiloglu et al. (2013).

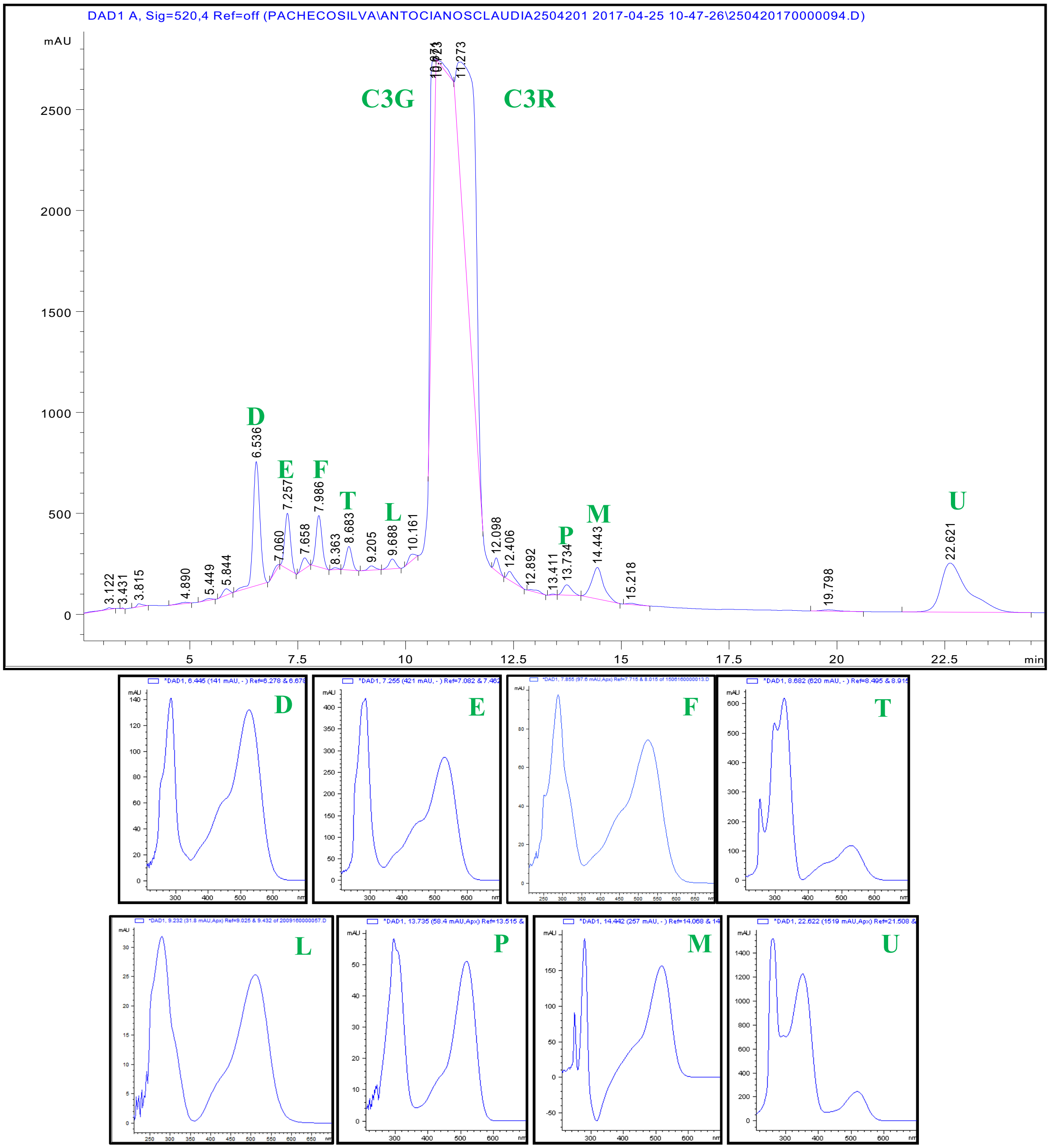

Supplementary Figure 2. HPLC profile of anthocyanins present in '98.99', 'Angeleno' and the 98Ang progeny. The shown profile was obtained by HPLC-DAD from a mixed sample (see section 2.3.3 in Materials and Methods). The anthocyanin detection in both records was at 520 nm, where the highest peaks corresponded to the C3G and C3R anthocyanins respectively. Those compounds that were not identified were named as "Compound X", where X corresponds to arbitrarily assigned letters. The image shows the peaks for C3G, C3R and 7 tentatively identified compounds (see Table 1).

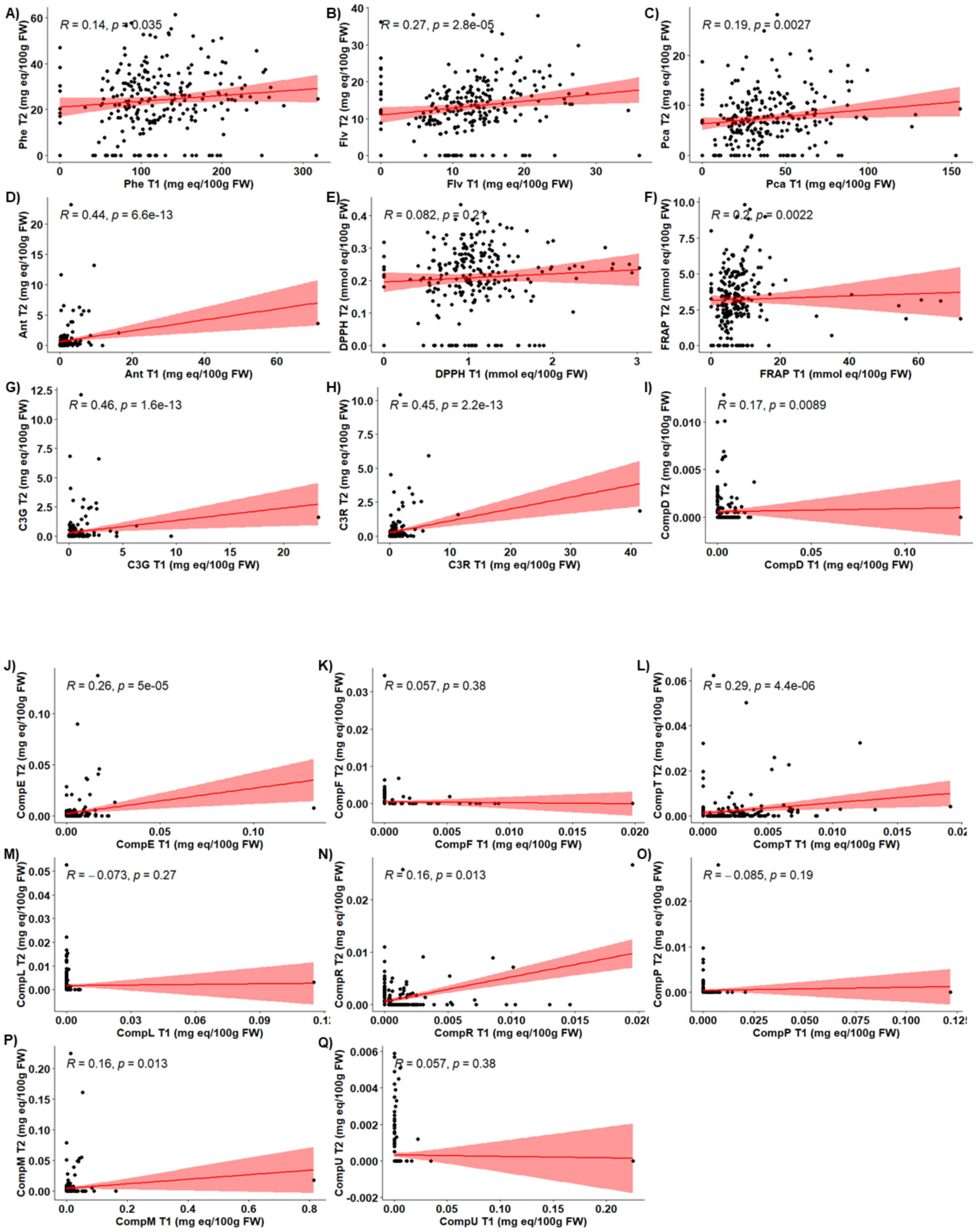

Supplementary Figure 3. Inter-season phenotypic correlation plot of PCC-traits in 98Ang for flesh tissue. Each histogram shows the Spearman correlations for the phenolic compound content traits of Asian plum samples. A: Phe, Total phenols; B: Flv, Total flavonoids; C: Pca, Total procyanidins; D: Ant, Total anthocyanins; E: DPPH antioxidant activity test; F: FRAP antioxidant activity test; G: C3G, Cyanidin 3-glucoside; H: C3R, Cyanidin 3-rutinoside; I: Comp. D; J: Comp. E; K: Comp. F; L: Comp. T; M: Comp. L; N: Comp. R; O: Comp. P; P: Comp. M; Q: Comp. U. In each graph, the axis corresponds to the concentration in the units belonging to each test (see Materials and Methods)

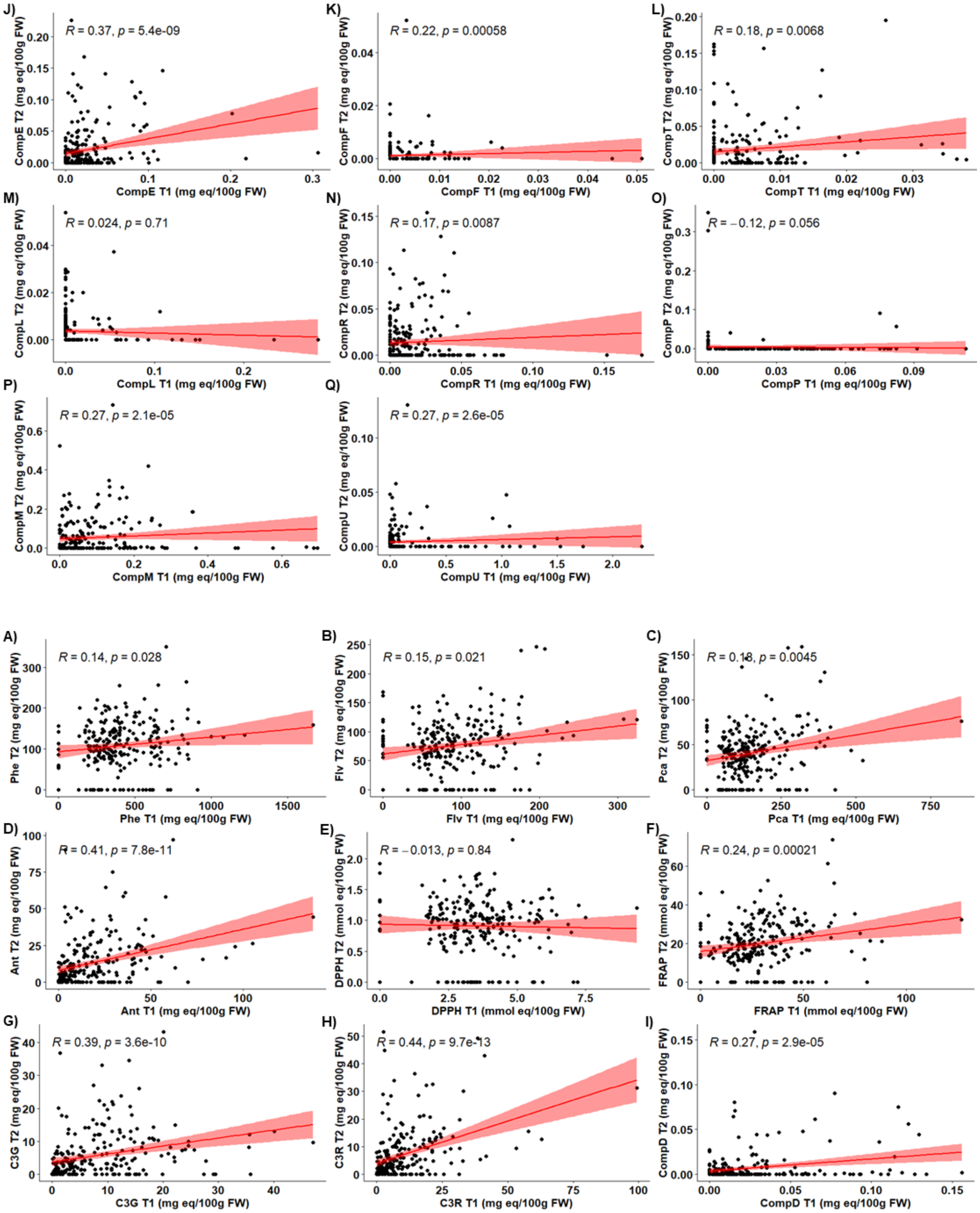

Supplementary Figure 4. Inter-season phenotypic correlation plot of PCC-traits in 98Ang for skin tissue. It presents the same description as Supplementary Figure 3.

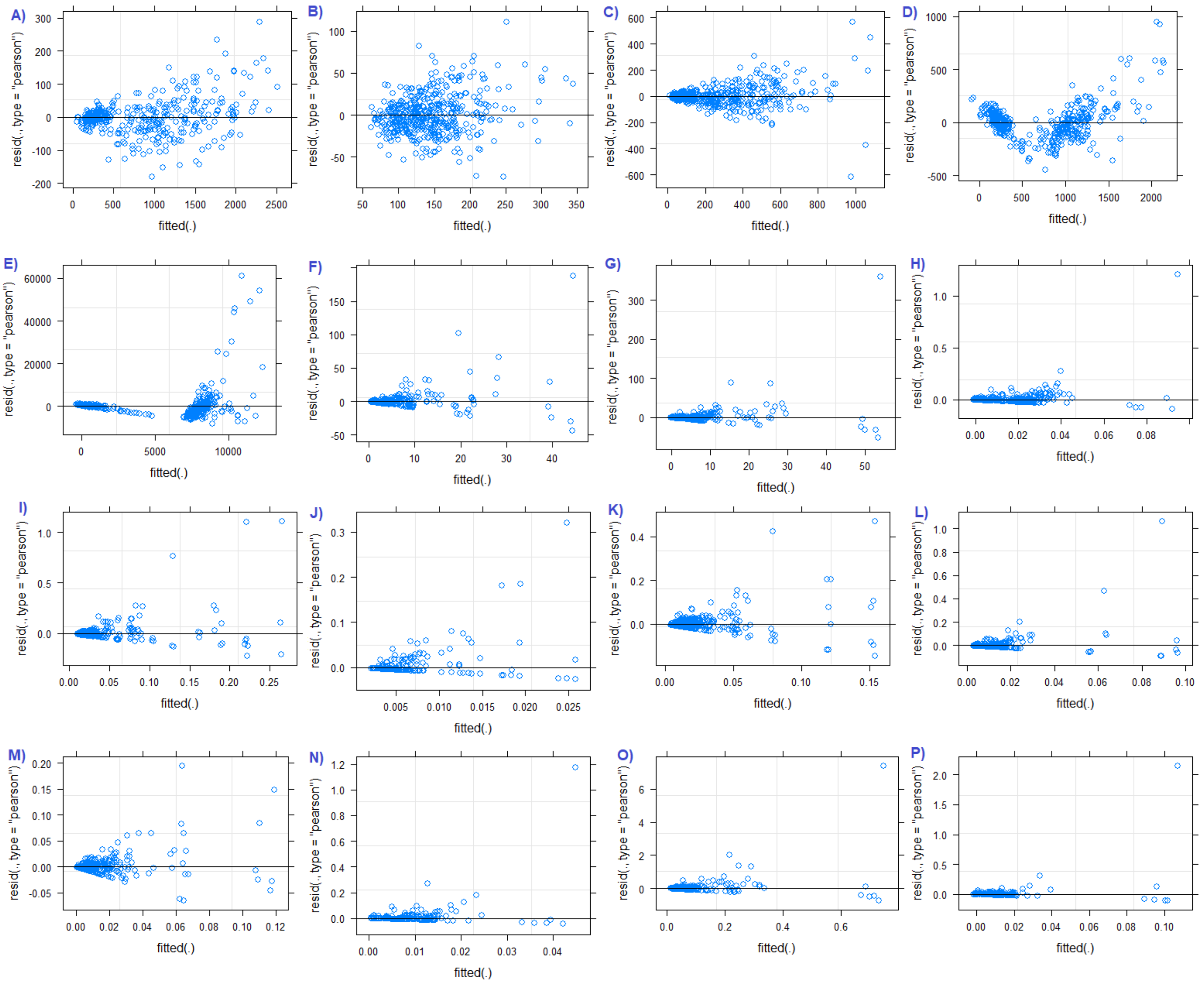

Supplementary Figure 5. MLM residual distribution graphs in flesh. To corroborate the fit of the linear mixed models generated, the dispersion of the residues the models for each PCC trait measured in flesh tissue was plotted. A valid linear mixed model must present a constant dispersion and without a clear trend in the residuals, that is, the residuals must present a random pattern. The adjusted values according to the generated model are indicated on the X axis, while the model residuals are distributed on the Y axis. A: Phe, Total phenols; B: Flv, Total flavonoids; C: Pca, Total procyanidins; D: DPPH antioxidant activity test; E: FRAP antioxidant activity assay; F: C3G, Cyanidin 3-glucoside; G: C3R, Cyanidin 3-rutinoside; H: Comp. D; I: Comp. E; J: Comp. F; K: Comp. T; L: Comp. L; M: Comp. R; N: Comp. P; O: Comp. M; P: Comp. U.

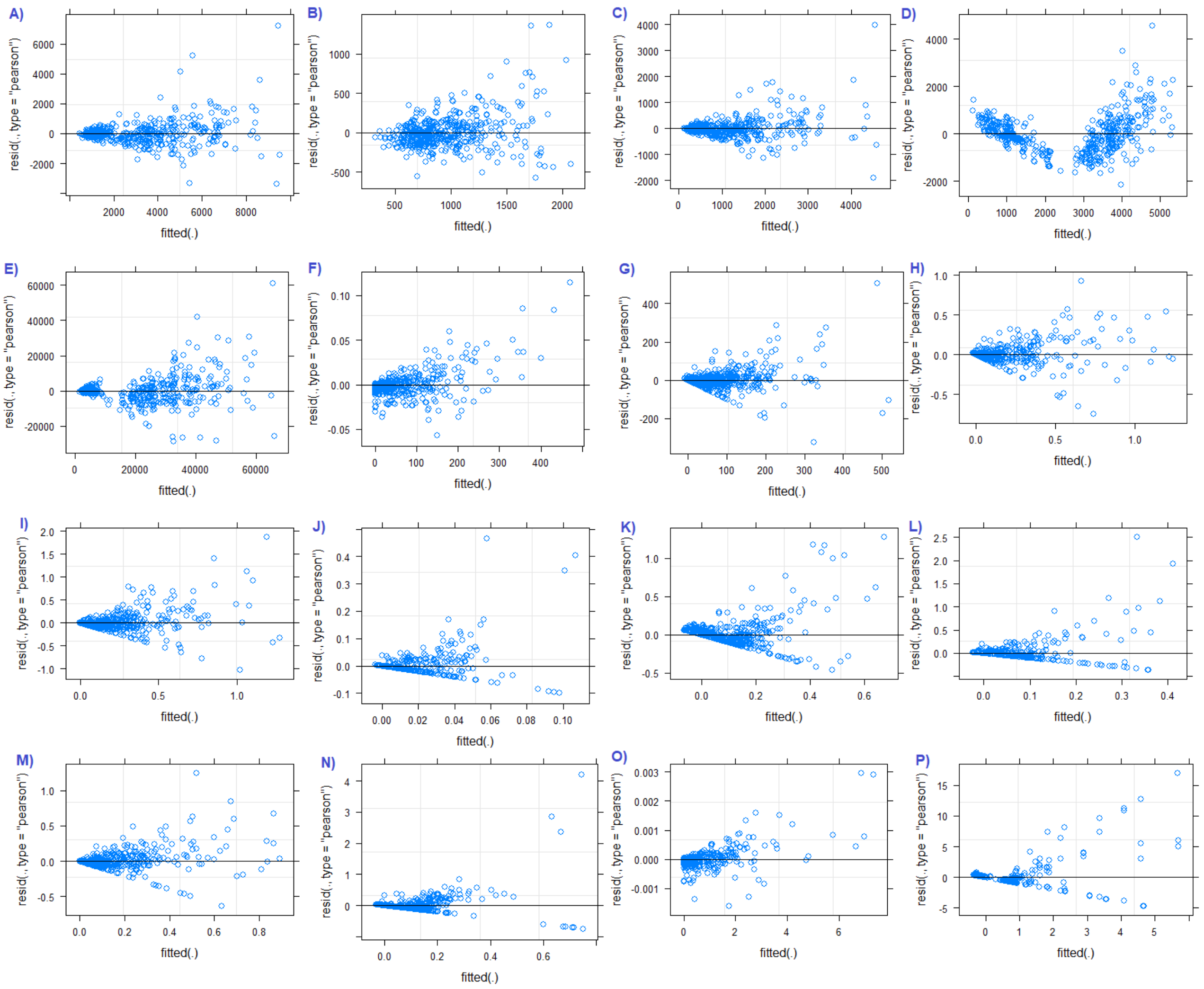

Supplementary Figure 6. MLM residue distribution graphs in skin. It presents the same description as Supplementary Figure 5.

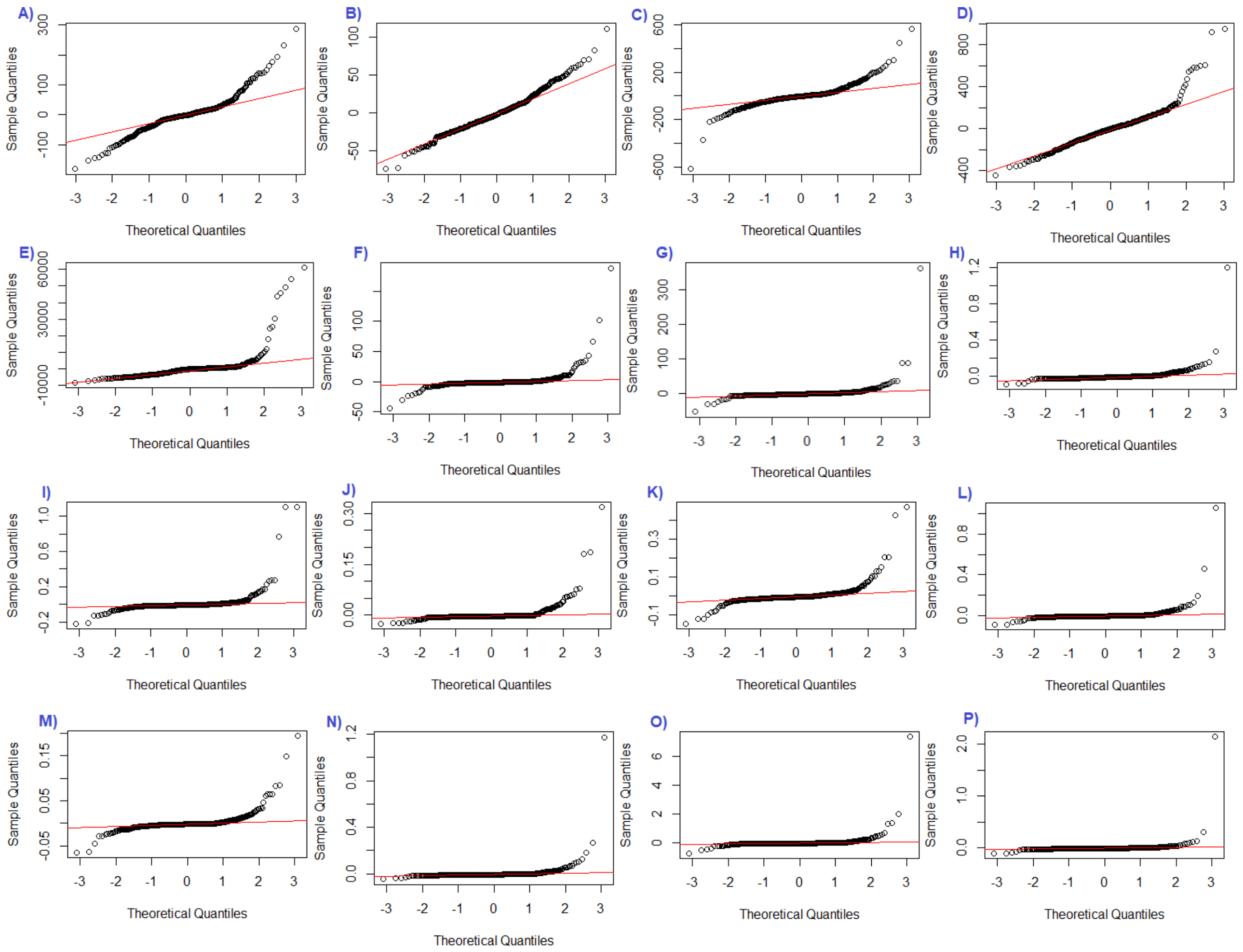

Supplementary Figure 7. Normal Q-Q plots of MLM residues in flesh. The normality verification of the generated MLM residuals is carried out with this type of graph, since it compares the distribution of the residuals obtained with the theoretical normal distribution, so that the residuals must be distributed as close as possible to the diagonal (line red) present in this type of graph to indicate that the residuals are normally distributed. The X axis indicates the theoretical normal distribution according to the generated model, while the Y axis indicates the distribution of the residuals. In both axes, the distribution is expressed in quartiles. A: Phe, Total phenols; B: Flv, Total flavonoids; C: Pca, Total procyanidins; D: DPPH antioxidant activity test; E: FRAP antioxidant activity assay; F: C3G, Cyanidin 3-glucoside; G: C3R, Cyanidin 3-rutinoside; H: Comp. D; I: Comp. E; J: Comp. F; K: Comp. T; L: Comp. L; M: Comp. R; N: Comp. P; O: Comp. M; P: Comp. U.

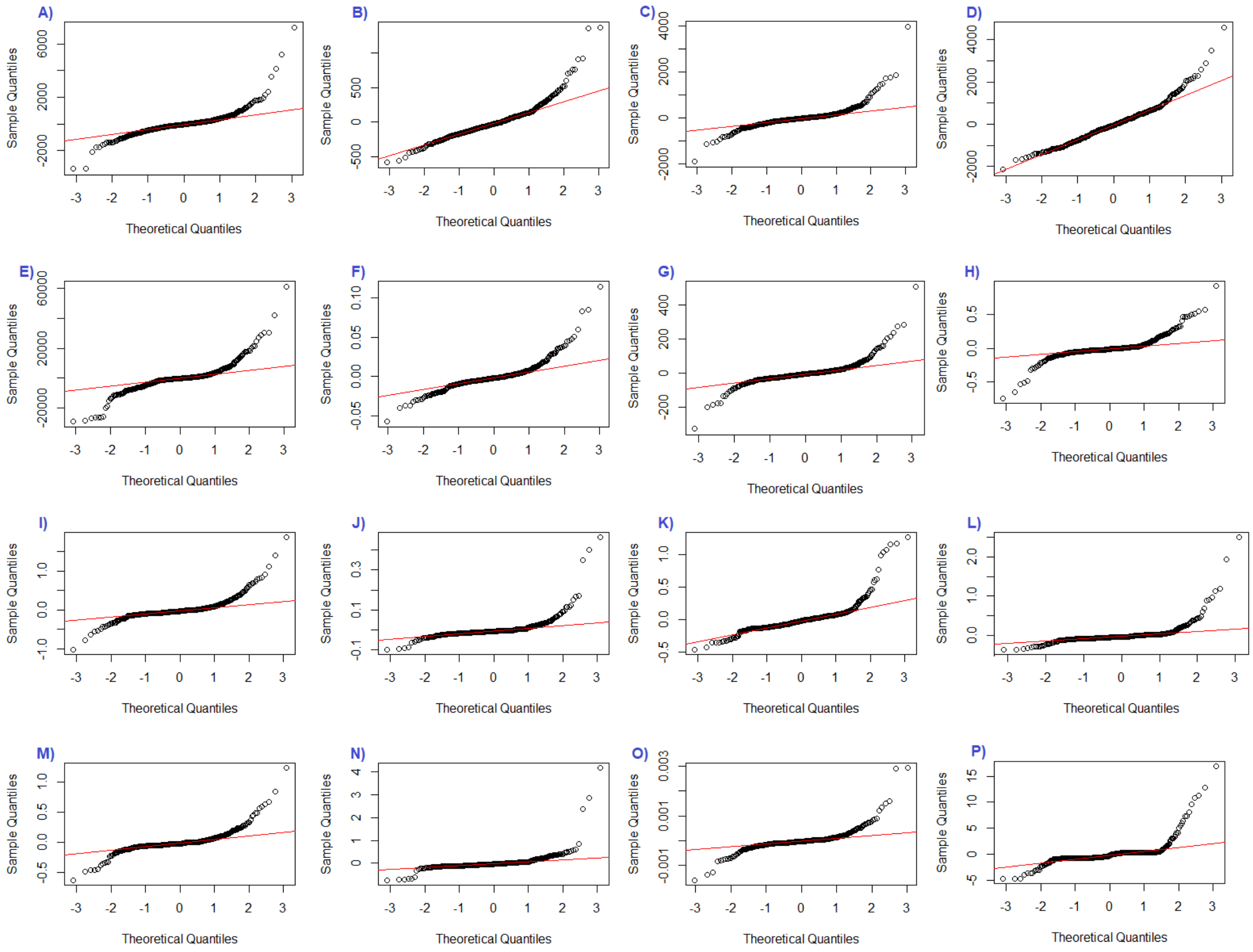

Supplementary Figure 8. Normal Q-Q plots of MLM residues in skin. It presents the same description as Supplementary Figure 7.
